# Inhibition of PDIs Downregulates Core LINC Complex Proteins, Promoting the Invasiveness of MDA-MB-231 Breast Cancer Cells in Confined Spaces In Vitro

**DOI:** 10.3390/cells13110906

**Published:** 2024-05-24

**Authors:** Natalie Young, Zizhao Gui, Suleiman Mustafa, Kleopatra Papa, Emily Jessop, Elizabeth Ruddell, Laura Bevington, Roy A. Quinlan, Adam M. Benham, Martin W. Goldberg, Boguslaw Obara, Iakowos Karakesisoglou

**Affiliations:** 1 Department of Biosciences, Durham University, Durham DH1 3LE, UK; natalie.young@durham.ac.uk (N.Y.); guizizhaoalbert@outlook.com (Z.G.); kleopatra.papa@ncl.ac.uk (K.P.); emilykjessop1@gmail.com (E.J.); elizabeth.ruddell@btinternet.com (E.R.); laura.bevington@yahoo.co.uk (L.B.); r.a.quinlan@durham.ac.uk (R.A.Q.); adam.benham@durham.ac.uk (A.M.B.); m.w.goldberg@durham.ac.uk (M.W.G.); 2 School of Computing, Newcastle University, Newcastle upon Tyne NE4 5TG, UK; suleiman213@gmail.com (S.M.); boguslaw.obara@newcastle.ac.uk (B.O.)

**Keywords:** intermediate filaments, lamin, LINC complex, nesprins, nuclear mechanics, nucleus, PDI, SUN1, SUN2, 16F16, PACMA31

## Abstract

Eukaryotic cells tether the nucleoskeleton to the cytoskeleton via a conserved molecular bridge, called the LINC complex. The core of the LINC complex comprises SUN-domain and KASH-domain proteins that directly associate within the nuclear envelope lumen. Intra- and inter-chain disulphide bonds, along with KASH-domain protein interactions, both contribute to the tertiary and quaternary structure of vertebrate SUN-domain proteins. The significance of these bonds and the role of PDIs (protein disulphide isomerases) in LINC complex biology remains unclear. Reducing and non-reducing SDS-PAGE analyses revealed a prevalence of SUN2 homodimers in non-tumorigenic breast epithelia MCF10A cells, but not in the invasive triple-negative breast cancer MDA-MB-231 cell line. Furthermore, super-resolution microscopy revealed SUN2 staining alterations in MCF10A, but not in MDA-MB-231 nuclei, upon reducing agent exposure. While PDIA1 levels were similar in both cell lines, pharmacological inhibition of PDI activity in MDA-MB-231 cells led to SUN-domain protein down-regulation, as well as Nesprin-2 displacement from the nucleus. This inhibition also caused changes in perinuclear cytoskeletal architecture and lamin downregulation, and increased the invasiveness of PDI-inhibited MDA-MB-231 cells in space-restrictive in vitro environments, compared to untreated cells. These results emphasise the key roles of PDIs in regulating LINC complex biology, cellular architecture, biomechanics, and invasion.

## 1. Introduction

In eukaryotes, the nuclear interior is linked to the cytoskeleton via a conserved macromolecular bridge, which spans the outer (ONM) and inner nuclear membranes (INM), known as LINC (linker of the nucleoskeleton and cytoskeleton) [1]. The LINC complex core consists of KASH (Klarsicht, ANC-1, Syne Homology) -domain and SUN (Sad1 and UNC-84) -domain proteins, which directly bind in the nuclear envelope (NE) lumen [1,2,3]. In mammals, spectrin repeat (SR) and KASH-domain containing proteins are known as nesprins (nuclear envelope spectrin repeat proteins) [4]. The spectrin repeats (SRs) occupy the N-terminal half or central part of these proteins, whilst the C-terminally located KASH-domain anchors them to the nuclear membrane [1,2,4]. In humans, four separate genes, namely *SYNE1*, *SYNE2*, *SYNE3*, and *SYNE4*, encode for Nesprin-1, Nesprin-2, Nesprin-3, and Nesprin-4 proteins, respectively [5,6]. Nesprins perform tailored interactions with cytoskeletal proteins via their unique N-terminal motifs. The largest isoforms of *SYNE1* and *SYNE2* encode Nesprin-1 (~1 MDa) and Nesprin-2 (~0.8 MDa) giant, respectively. Both bind F-actin through their N-terminal actin-binding domain (ABD), which contains two calponin homology domains (CH1 and CH2) [7,8]. Nesprin-3α interacts with the cytolinker plectin via its first spectrin repeat (SR1), recruiting intermediate filaments (IFs) to the ONM [9]. Nesprin-4 associates indirectly with microtubules (MTs) by recruiting kinesin-1, which facilitates nuclear positioning [10]. Nesprin-1 and -2 proteins localise to both the ONM and INM [11,12]. In contrast, Nesprin-3 and Nesprin-4 localise exclusively to the ONM [9,10]. The NE localisation of nesprins requires the presence of SUN-domain and nuclear lamina proteins [11]. In human HaCaT keratinocytes, SUN1 silencing alone is sufficient to alter the Nesprin-2 localisation from the nucleus [2]. However, in HeLa cells, the Nesprin-2 NE pattern changed only when both SUN1 and SUN2 proteins were depleted [1]. Similar observations were made in mouse mutants, where Nesprin-1 NE localisation was disrupted only in SUN1/SUN2 double knockout animals [13]. 

SUN-domain proteins are evolutionarily conserved INM proteins. The human genome contains five SUN-domain genes; the ubiquitously expressed *SUN1* and *SUN2*, as well as *SUN3*, *SUN4* (*SPAG4*), and *SUN5* (*SPAG4L*), which exhibit a testis-specific expression pattern [2,14,15,16,17,18,19]. The hallmark of the protein family is the conserved C-terminal SUN-domain, which is preceded by a less-conserved coiled-coil segment, residing in the NE lumen. The N-terminus of SUN-domain proteins faces the nuclear interior, associating with nuclear IFs (i.e., lamins), DNA-binding proteins, and chromatin, while the central section contains at least one transmembrane domain [20]. Extensive literature highlights that SUN-domain proteins form higher-order structures. The emerging understanding of LINC complexes reveals a complex landscape, characterised by inter-species differences and the potential presence of structurally and functionally diverse LINC complexes within a single cell. Vertebrate SUN1 forms monomers, dimers, and tetramers, and its oligomerisation involves the coiled-coil region and interchain disulphide bonds [21]. In addition, SUN1 associates with SUN2 via its pair of coiled-coils and exhibits restricted protein dynamics in iFRAP (inverse fluorescence recovery after photobleaching) experiments. Importantly, distinct SUN1 protein pools at the sub-cellular level have been identified using confocal microscopy [21]. The *D. discoideum* SUN1 C-terminus forms monomers, homodimers, and trimers in vitro, involving its central coiled-coil region [22]. X-ray crystallography of SUN2’s SUN-domain revealed a homo-trimeric structure involving the coiled-coil region [23,24]. Crystallographic analysis of SUN-domain and Nesprin-1/Nesprin-2 KASH-peptides unveiled a hexameric complex (3:3 SUN-domain to KASH-domain stoichiometry), with the KASH-binding site created by adjacent SUN-domain protomers. In addition, an intrachain disulphide bond was found between the amino acid residues Cys601 and Cys705 of the SUN-domain, along with an additional heteromeric inter-chain disulphide bond with SUN2 residue Cys563 (UniProt identifier: Q9UH99) and a conserved Nesprin-2 giant KASH-domain cysteine (Cys6862; UniProt identifier: Q8WXH0) [23]. Further insights into SUN-domain protein oligomerisation and KASH-domain binding were obtained through the crystallisation and biochemical analysis of additional SUN2 recombinant proteins. These proteins included longer fragments of the SUN2 C-terminus, containing the SUN-domain and the coiled-coil 1 (CC1) and 2 (CC2) sub-domains, or single fragments of the coiled-coil region [25]. The resulting model suggests that SUN2 may also exist as an inactive monomer. This inactive state, characterised by the SUN-domain’s inability to bind to KASH-domains, is attributed to its association with CC2. Binding to KASH-domains (referred to as the active state) occurs only when CC1 is present. The presence of CC1 initiates protein trimerisation and activates the SUN-domain [25]. More recent crystallographic evidence of SUN-KASH complexes also indicates the presence of head-to-head assemblies, involving two canonical (i.e., 3:3) SUN–KASH structures, resulting in a single LINC complex dodecamer [26]. Despite conflicting structural evidence, the consensus is that LINC complexes can form supramolecular structures due to the ability of SUN-domain proteins to oligomerise through their coiled-coil domain and by interchain disulphide bonds. Hence, this potentially implies the regulatory involvement of PDI (protein disulphide isomerase) family members in the tertiary structure of SUN-domain proteins and their covalent interactions with KASH-domain proteins, via their ability to mediate disulphide bond exchange.

PDIs are abundant and multifunctional oxireductases, belonging to the thioredoxin (TRX) superfamily of proteins [27]. The PDI family comprises 21 members, which reside primarily in the endoplasmic reticulum (ER), playing key roles in ER-proteostasis. Specifically, these enzymes catalyse disulphide (S–S) bond formation (oxidation), breakage (reduction), and rearrangement (isomerisation). Furthermore, selective PDI members exhibit both molecular chaperone and anti-chaperone activities [27,28].

PDIA1, the most studied member of the PDI family, is archetypal, widely expressed, and contains two redox-active TRX-like domains [29]. PDIA1 is frequently overexpressed in breast tumour tissues [30]. Compared to primary breast carcinoma cells, PDIA1 is significantly upregulated in the cells that have metastasised to the axillary lymph nodes [31,32]. In the highly invasive triple-negative breast cancer cell line, MDA-MB-231, PDIA1 is the dominant PDI paralogue, followed by PDIA3 [33]. Interestingly, the PDIs, PDIA1, ERp44, and PDIA3 (also known as ERp57), promote the anchorage-independent proliferation of breast cancer cells, a feature commonly found in transformed cells of solid tumours, facilitating mammosphere formation in vitro [34]. The levels of PDIA3 (ERp57) are upregulated in invasive breast tumours [35,36,37,38]. Moreover, PDIA3 expression in fibroblasts enhances the pro-migratory properties of breast cancer cells in vitro [39]. While PDIs tend to be overexpressed in cases of advanced breast cancer, the LINC complex proteins Nesprin-2, SUN1, SUN2, and Lamin A/C are downregulated in human breast cancer cells [40]. Interestingly, SUN2 operates as a tumour suppressor, counteracting the Warburg effect, and exhibits reduced expression levels in lung cancer [41]. However, the functional relationship between PDIs and SUN-domain proteins, and the LINC complex in general, remains unknown. 

The current study investigates the relationship between the LINC complex and PDI biology in breast cancer cell progression. Specifically, it examines whether SUN1 and SUN2 disulphide bond-mediated oligomerisation is altered in non-tumorigenic MCF10A breast epithelia compared with invasive MDA-MB-231 cells. Super-resolution microscopy imaging in the presence and absence of reducing agents provides novel insights into the spatial distribution of SUN2 structures across the MCF10A nucleus. The regulatory role of PDIs in LINC complex protein expression, localisation, and perinuclear cytoskeletal architecture is established using two PDI inhibitors; 16F16 and PACMA31. While both compounds are small, cell-permeable, and irreversible inhibitors of PDIs, they differ in their PDI substrate specificity; 16F16 preferentially inhibits PDIA3 over PDIA1 [42], while PACMA31 exhibits a broader range of PDI inhibition (e.g., PDIA1, PDIA3, PDIA4, PDIA6, TXNDC5), with stronger inhibitory effects observed for PDIA1 [43,44]. By examining the effects of PDI-inhibition on the invasiveness of MDA-MB-231 cells using space-restrictive porous inserts, novel mechanistic insights are provided, involving fundamental cell stiffness determinants (e.g., F-actin, IFs, and lamins) that may aid the development of robust therapeutic strategies involving PDI inhibitors for breast cancer treatment.

## 2. Materials and Methods

### 2.1. MCF10A and MDA-MB-231 Cell Culture

MCF10A cells (CRL-10317) were obtained from the American Type Culture Collection (ATCC, Manassas, VA, USA), while the MDA-MB-231 cells (item number 92020424) were acquired from the European Collection of Authenticated Cell Cultures (ECACC, Salisbury, UK). These cell lines, representing non-tumorigenic breast epithelia (MCF10A) and triple-negative breast cancer (MDA-MB-231), respectively, were cultured routinely as cell monolayers in a humidified cell culture incubator, at 37 °C and 5% CO_2_ (PHCbi, Loughborough, UK). Both cell lines were maintained in phenol red-free media. The conditions for cell culture were as follows. 

MCF10A cells: DMEM/F-12 medium (Sigma-Aldrich, Gillingham, UK) supplemented with 5% (*v*/*v*) horse serum (Invitrogen, Loughborough, UK), 2 mM L-Glutamine (Sigma-Aldrich, Gillingham, UK), 1% penicillin–streptomycin (Sigma-Aldrich, Gillingham, UK), 500 ng/mL hydrocortisone (Sigma-Aldrich, Gillingham, UK), 0.01 mg/mL insulin (Sigma-Aldrich, Gillingham, UK), 100 ng/mL cholera toxin (Sigma-Aldrich, Gillingham, UK), and 20 ng/mL epidermal growth factor (Peprotech, ThermoFisher Scientific, Cramlington, UK). 

MDA-MB-231 cells: DMEM medium containing 4.5 g/L glucose (Corning, Flintshire, UK), supplemented with 10% fetal bovine serum (FBS; Sigma-Aldrich, Gillingham, UK), 2 mM L-Glutamine (Sigma-Aldrich, Gillingham, UK) and 1% penicillin–streptomycin (Sigma-Aldrich, Gillingham, UK). Cells were routinely passaged with the use of a trypsin–EDTA solution (Sigma-Aldrich, Gillingham, UK). First, the media were removed, then the cells were washed twice with DPBS (Dulbecco’s phosphate-buffered saline; Gibco, ThermoFisher Scientific, Cramlington, UK). This was followed by the addition of trypsin–EDTA to the cell monolayer. The cells were incubated at 37 °C, 5% CO_2_ (MCF10A, 20 min; MDA-MB-231, 10 min), with full cell detachment determined with the use of the Zeiss Telaval 31 light microscope (Carl Zeiss Ltd, Cambridge, UK). Suspended cells were mixed with the medium and collected into a falcon tube, before being centrifuged to pellet the cells (1000× *g*, 5 min; Fisherbrand^TM^ Centrifuge GT2, ThermoFisher Scientific, Cramlington, UK). The medium was removed carefully, so as not to disturb the pellet, and cells were subsequently resuspended in fresh medium. Cell numbers were calculated with the use of the Countess 3^TM^ automated cell counter (ThermoFisher Scientific, Cramlington, UK).

To pharmacologically inhibit members of the PDI enzyme family, the compounds 16F16 and PACMA31 (Sigma-Aldrich, Gillingham, UK) were utilised. Both compounds were initially dissolved in DMSO (Dimethyl Sulfoxide) to prepare stock solutions. Subsequently, the drugs were diluted in MDA-MB-231 cell media to a final concentration of 5 μM for 16F16 and 2.5 μM for PACMA31. The cells were then incubated with the prepared media for 24 h at 37 °C and 5% CO_2_ in a humidified cell incubator.

### 2.2. Immunofluorescence Staining and Microscopy Imaging

MCF10A and MDA-MB-231 cells were cultured on glass coverslips (11 mm, LaboQuip, London, UK) until reaching ~60%–70% confluency. The coverslips were rinsed twice in 1× BRB80 (80 mM K-PIPES (ThermoFisher Scientific, Cramlington, UK), 1 mM MgCl_2_ (Sigma-Aldrich, Gillingham, UK), 1 mM EGTA (Sigma-Aldrich, Gillingham, UK)), adjusted to pH 6.8 with KOH (ThermoFisher Scientific, Cramlington, UK)), then concurrently fixed and permeabilised (3.7% Formaldehyde, 0.5% Triton X-100 in 1× BRB80) for 20 min within a humidified cell incubator set at 37 °C, 5% CO_2_. The fixed cells were subsequently rinsed with PBS (phosphate-buffered saline; Severn Biotech, Kidderminster, UK), then blocked with phosphate-buffered gelatine (PBG), containing 0.1% bovine serum albumin (BSA; Sigma-Aldrich, Gillingham, UK), 0.1% fish gelatine (Sigma-Aldrich, Gillingham, UK), and 0.1% Triton X-100 (ThermoFisher Scientific, Cramlington, UK), for 1 h at room temperature. Cells were labelled with primary antibodies (as listed in Table S1), which were diluted in PBG solution, within a humidified chamber for 1 h at room temperature, followed by three washes with PBS (each washing step lasting 10 min). The cells were subsequently incubated with the corresponding secondary antibodies (as listed in Table S2), diluted in PBG, followed by three washes in PBS (each wash lasting 10 min) in the dark. The samples were counterstained with DAPI solution (2 μg/mL, in PBS; Sigma-Aldrich, Gillingham, UK) for 5 min at room temperature in the dark, to stain the DNA. Afterwards, the coverslips were mounted onto slides (LaboQuip, London, UK) using VECTASHIELD^®^ Antifade Mounting Medium (2bscientific, Bicester, UK). The resulting specimens were analysed using both the Axioskop 40 epifluorescence microscope (Carl Zeiss Ltd, Cambridge, UK), as well as the Zeiss LSM 880, Axio Observer Z1 microscope system (Carl Zeiss Ltd., Cambridge, UK). 

For the super-resolution imaging of SUN2 (Figure 2), MCF10A and MDA-MB-231 cells were cultured on high-precision coverslips (22 mm × 22 mm, LaboQuip, London, UK). Upon reaching 60–70% confluency, the cells were treated with 1 μM DTT or media for 30 min. Following treatment, the coverslips were processed for immunofluorescence analysis, as outlined above. The resulting samples were imaged using a Zeiss LSM 880, Axio Observer Z1 microscope system (Carl Zeiss Ltd, Cambridge, UK), using the following imaging parameters: 

Laser specifications and settings: A 594 nm helium-neon (He/Ne) laser was utilised to excite the Alexa Fluor 568 fluorophores of the sample. The laser was set to an output of 2% intensity, master gain: 1000, without digital offset. Objective: Plan-Apochromat 63x/1.4 Oil DICM27. Main beam splitter: MBS 488/594. Emission filter: BP570-620 + LP645. Detector type: Airyscan. Airyscan Mode: Airyscan SR. Image size (pixels): Mostly 496 × 496 with a pixel dwell time of 2.02 μs. Pixel size: Calculated according to the Nyquist theorem, determined via Zen Black optimal pixel size setting, resulting in an image pixel size of 0.05 μm × 0.05 μm × 0.21 μm (X,Y,Z). Imaging settings: The images were acquired as Z-stacks, with a single Z-stack thickness of 0.21 μm. Imaging processing and software: Airyscan images were processed with filtering, pixel reassignment, and deconvolution in Zen Black 2.1 (Version 14.0.0.0), set at automatic filter strength with typical values ranging between 6.6-7.1.

### 2.3. EdU Cell Proliferation Assay

To assess cell proliferation, the Click-iT^®^ EdU imaging kit (ThermoFisher Scientific, Cramlington, UK) was employed, according to the manufacturer’s guidelines. Briefly, 20 μM EdU diluted in fresh media was added directly to MDA-MB-231 cells in a 1:1 ratio with the existing media, giving a final concentration of 10 μM EdU. The cells were then incubated in a humidified cell incubator at 37 °C with 5% CO_2_ for 45 min. Afterwards, the cells were fixed in 4% paraformaldehyde PBS buffer at room temperature for 20 min. Subsequently, the cells were permeabilised with 0.5% Triton X-100 in PBS for 10 min at room temperature. The coverslips were then rinsed in a 3% BSA/PBS solution (Sigma-Aldrich, Gillingham, UK), followed by incubation in 500 μL of Click-iT^®^ reaction cocktail at room temperature for 30 min. The cells were rinsed in 3% BSA/PBS and then incubated at room temperature for 5 min with 2 µg/mL DAPI (Sigma-Aldrich, Gillingham, UK) to label nuclei, followed by a final wash with 3% BSA/PBS. Finally, the coverslips were mounted on glass slides (LaboQuip, London, UK) and imaged following the procedure described in Section 2.2.

### 2.4. SDS-PAGE and Western Blotting

**Figure 1:**
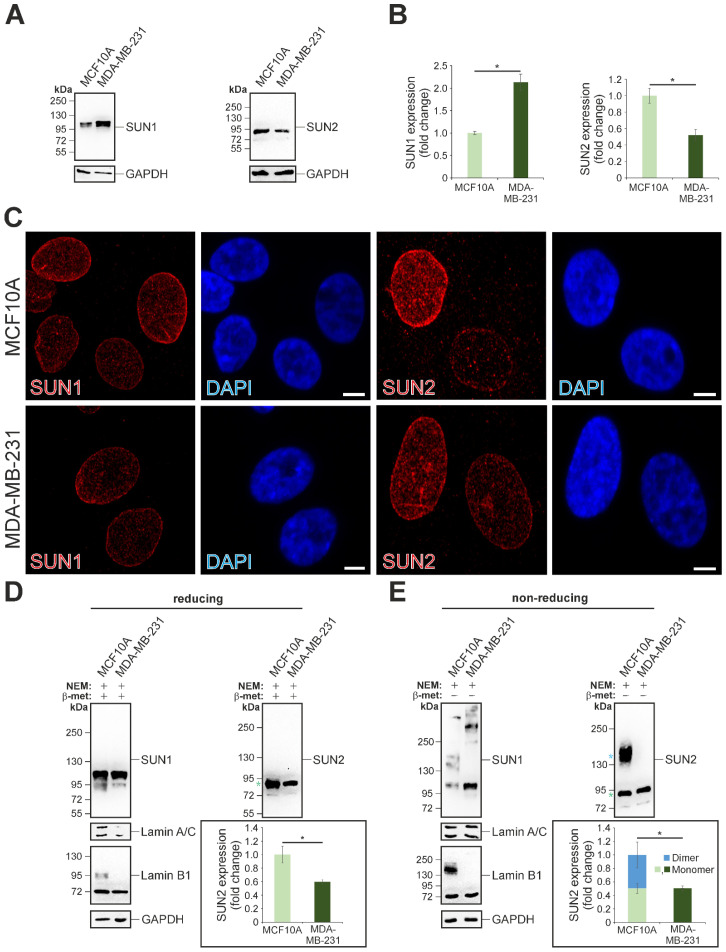
In MDA-MB-231 cells, the intermolecular disulphide bonds of SUN1, SUN2, and Lamin B1 are altered compared to MCF10A cells. (**A**) Determination of SUN1 and SUN2 protein expression using quantitative Western blotting. GAPDH protein expression levels indicate equal protein loading of samples. Cell lysates were analysed using 10% SDS-PAGE gels. (**B**) Densitometric analysis of protein expression is shown in panel A. The data are represented as the mean ± SEM (standard error of the mean), *n* = 3. Statistical significance was assessed using a Student’s unpaired *t*-test, the asterisk (*) denotes significance (*p* ≤ 0.05). (**C**) Immunofluorescence confocal microscopy examination of SUN1- and SUN2-stained cells. Scale bar: 5 μm. (**D**) Western blot analysis of cell lysates under reducing conditions, treated with the alkylating agent N-ethylmaleimide (NEM) and a reducing agent (1% β-mercaptoethanol [β-met]), following separation by SDS-PAGE using 4–12% gradient polyacrylamide gels. GAPDH is used as a protein loading control. Quantitative analysis of SUN2 expression is represented as the mean ± SEM, *n* = 3. Statistical significance was determined employing a Student’s unpaired *t*-test and significance is indicated by the asterisk (*) at *p* ≤ 0.05. (**E**) Western blot analysis of cell lysate protein samples under non-reducing conditions, without β-mercaptoethanol (β-met). Note the presence of disulphide-linked homodimers for Lamin B1 and SUN2 in MCF10A, but not in MDA-MB-231 cells. The graph represents SUN2 dimer and monomer expression in MCF10A and MDA-MB-231 cells. The expression data are represented as the mean ±SEM, *n* = 3. Statistical significance (*, *p* ≤ 0.05) was assessed using a Student’s unpaired *t*-test.

Cell lysate preparation, SDS-PAGE (sodium dodecyl sulfate polyacrylamide gel electrophoresis), and Western blotting were conducted following the experimental procedures described previously [45]. The primary and secondary antibodies used are detailed in Tables S3 and S4, respectively. To examine the oligomerisation status of SUN1, SUN2, Lamin A/C, and Lamin B1, cell lysates containing 20 mM N-Ethylmaleimide (NEM) were prepared under non-reducing (omission of β-mercaptoethanol) and reducing (1% β-mercaptoethanol) conditions. Unlike the conventional 99 °C denaturing step, which is standard for most Western blot data (except for Figure 1D,E), the samples in this case were incubated for 4 min at 50 °C. This deviation was based on previous observations of SUN1 protein separation using SDS-PAGE [21]. For Western blot detection, Immobilon^®^-P PVDF transfer membranes (Merck Millipore, Watford, UK) were incubated with the Amersham ECL Prime Western blotting detection reagent (GE Healthcare Life Sciences, Little Chalfont, UK). Following substrate removal, the membranes were subjected to CL-Xposure Film exposure (ThermoFisher Scientific, Loughborough, UK) in the dark room, and developed using an X-OMAT X-ray developer (Kodak, Hertfordshire, UK). Alternatively, the Clarity Western ECL substrate (BioRad, Watford, UK) was used and the ECL signals were detected using an iBright imaging system (ThermoFisher Scientific, Loughborough, UK). Densitometry was employed to quantify the relative protein expression levels, with the data normalised to the corresponding loading controls (e.g., α-actin, β-tubulin, or GAPDH). The analysis was conducted using Fiji.

### 2.5. 2D Scratch Wound Assay

MDA-MB-231 cells were cultured to 100% confluency in 12-well cell culture plates (Starlab, Milton Keynes, UK). Prior to the start of the experiment, cells were incubated in serum-free media for 24 h in a humidified cell incubator (PHCbi, Loughborough, UK) at 37 °C, 5% CO_2_. Following incubation, a single scratch at the centre of each well was made to the cell monolayer using a 200 μL pipette tip (Starlab, Milton Keynes, UK). Cells were imaged using a Cell Observer microscope (Carl Zeiss Ltd, Cambridge, UK); objective: EC Plan-Neofluar 10×/0.3 Ph1, equipped with an environmental chamber set at 37 °C and 5% CO_2_, with a series of images taken at an interval of 15 min for 24 h. 

### 2.6. Cell Polarisation Assay

MDA-MB-231 cells were cultured on glass coverslips (11 mm, LaboQuip, London, UK) until reaching 100% confluency. A single scratch was made down the centre of the cell monolayer, using a 200 μL pipette tip (Starlab, Milton Keynes, UK). The cells were then incubated at 37 °C, 5% CO_2_ for 2 h to allow cell polarisation. Subsequently, the coverslips were fixed, permeabilized, and blocked, as described in Section 2.2. Primary antibody incubation against the Golgi apparatus component GM-130 (BD Biosciences, Wokingham, UK) was carried out, following the protocol outlined in Section 2.2, to highlight the migration direction of each cell. Concurrent secondary antibody and TRITC-phalloidin (20 ng/mL; Sigma-Aldrich, Gillingham, UK) labelling were then carried out, as described in Section 2.2. The coverslips were subsequently imaged using an Axioskop 40 epifluorescent microscope (Carl Zeiss Ltd., Cambridge, UK). 

### 2.7. Space Restrictive Cell Migration/Chemotaxis Assay

Prior to the execution of the restrictive migration assay, MDA-MB-231 cells were serum starved for 24 h in a humidified incubator at 37 °C, 5% CO_2_. After 24 h, the cells were collected and counted using a Countess 3^TM^ automated cell counter (ThermoFisher Scientific, Cramlington, UK). The 96-well cell migration/chemotaxis plate (ab235693, 5 μm pore, Abcam, Cambridge, UK) was disassembled. Medium containing 10% FBS was added to the wells of the bottom plate, with the FBS serving as a chemoattractant for the serum starved cells. The plate was reassembled and cells were plated into the wells of the top chamber, at a density of 50,000 cells per well. The cells were treated with the PDI inhibitors, either PACMA31 or 16F16 (Sigma-Aldrich, Gillingham, UK), at concentrations ranging from 1 to 10 μM. Alternatively, the cells were incubated with media (control) or 0.025% DMSO only (vehicle), and the 96-well plate was then incubated for 24 h at 37 °C, 5% CO_2_. After 24 h, the plate was disassembled, media aspirated from the top plate, and excess cells were removed using a cotton swab. The bottom chamber was centrifuged at 1000× *g* for 5 min at room temperature. After aspirating the media from the wells of the bottom chamber, the cells were washed using the supplied wash buffer. The plate was centrifuged again at 1000× *g* for 5 min at room temperature. A cell dye/cell dissociation solution was prepared in a 1:10 ratio and added to the wells of the bottom chamber. The plate was reassembled and incubated for 1 h at 37 °C and 5% CO_2_. Following incubation, the bottom plate was read at excitation/emission 530/590 nm using a BioTek Synergy HT plate reader (BioTek, Swindon, UK). A standard curve was generated simultaneously using known cell numbers (50,000; 25,000; 12,500; 6250; 3125; 1562; and 781), to determine the precise number of migrated cells under each condition.

### 2.8. MTT Cytotoxicity Assay

MDA-MB-231 cells were plated into the wells of a 96-well plate (Starlab, Milton Keynes, UK), at a concentration of 4000 cells per well. The cells were incubated in a humidified incubator at 37 °C and 5% CO_2_ for 24 h to allow the cells to adhere. 

After 24 h, the media was replaced with either complete media (containing FBS), or, to address the role of proliferation, serum-free media (without FBS), and the cells were given a further 24-h incubation at 37 °C, 5% CO_2_. After 24 h, the cells were treated with the PDI inhibitors, either PACMA31 or 16F16 (Sigma-Aldrich, Gillingham, UK), at concentrations ranging from 1 to 10 μM, or incubated with their subsequent controls, either media (control) or 0.025% DMSO only (vehicle). The plate was placed in a cell culture incubator and incubated for an additional 24 h. Following incubation, 0.5 mg/mL MTT (3-(4,5-Dimethylthiazol-2-yl)-2,5-Diphenyltetrazolium Bromide (ThermoFisher Scientific, Cramlington, UK) was added to each well. The cells were then left to incubate for a further 3 h at 37 °C and 5% CO_2_. Subsequently, the MTT/media liquid was carefully aspirated from each well and the formazan crystals were dissolved in DMSO (Dimethyl Sulfoxide; ThermoFisher Scientific, Cramlington, UK). The absorbance was then measured at 570 nm, using a microplate reader (BioTek ELx800, Swindon, UK). 

### 2.9. SUN2 Bioimage Analysis

**Figure 2:**
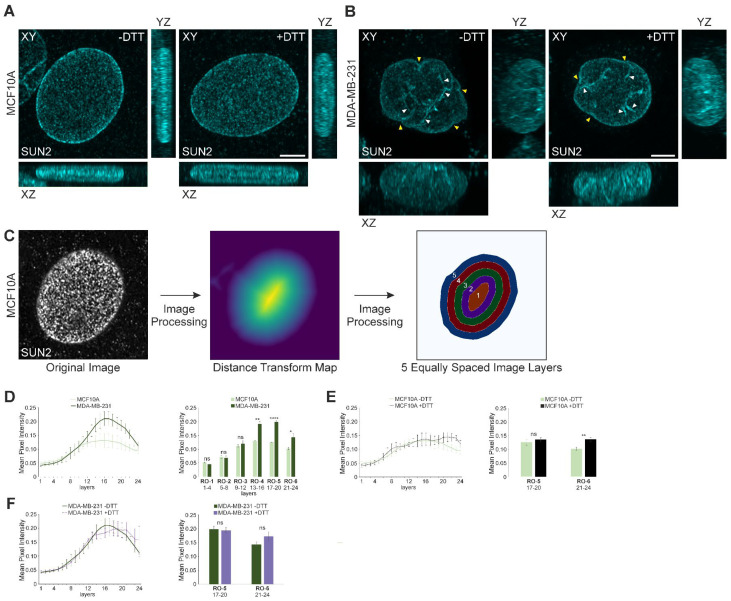
The organisation of SUN2 differs between MCF10A and MDA-MB-231 nuclei, with DTT treatment affecting only the outermost 3D layers of MCF10A nuclei. (**A**,**B**) The micrographs depict representative (**A**) MCF10A and (**B**) MDA-MB-231 nuclei immunostained for SUN2, without (−) and with (+) a 30-min pre-treatment of 1 μM DTT (dithiothreitol). Maximum intensity projections (frontal XY, transverse XZ, and sagittal YZ) were produced from Z-stack microscopy images generated using a Zeiss LSM 880 Airyscan confocal laser microscope. Scale bar: 5 μm. White arrowheads indicate nuclear indentations and SUN2 aggregates. Yellow arrowheads indicate nuclear shape irregularities. Scale bar: 5 μm. (**C**) Simplistic layout of SUN2 immunofluorescence confocal microscopy image analysis. A representative original Z-stack microscopy image of SUN2 staining from panel A (-DTT) is shown. The 3D-image was subjected to image processing, which included the generation of a distance transform map (read the M and M section for more details). The distance transform map represents the 3D spatial distance of every SUN2 pixel within the 3D nucleus (SUN2 voxel) from the nuclear periphery. Lighter colours depict longer distances (closer to the nuclear centre), while darker colours denote shorter distances (closer to the nuclear periphery). Subsequently, the 3D-image was divided into equally-spaced concentric 3D layers. To facilitate comprehension, only five layers (out of the 24 used) are displayed in the figure, which represents a cross-section of the 3D-image, showcasing the concentric arrangement of these layers in 2D. Layer 1 is positioned at the nuclear centre, while layer 5 represents the outermost 3D layer. (**D**) The average pixel intensity values for SUN2 staining in MCF10A and MDA-MB-231 nuclei were assessed in each of the 24 evenly-spaced 3D layers (left panel). The histogram (right panel) provides a summary of the mean pixel intensity for SUN2 in MCF10A and MDA-MB-231 nuclei within specific regions of interest (RO, highlighted in bold). Each RO corresponds to four equally-spaced 3D layers, which are denoted below the RO X-axis labels. RO-1 represents the innermost region of interest at the nuclear center, while RO-6 represents the nuclear region furthest away from RO-1. (**E**,**F**) The average pixel intensity of SUN2-stained MCF10A (**E**) and MDA-MB-231 (**F**) nuclei, without (−) and with (+) 1 μM DTT (Dithiothreitol) 30-min pre-treatment for 24 uniform concentric 3D layers, is shown (left panel). Representation of the data for specific nuclear regions of interest five (RO-5; representing nuclear outside layers 17–20) and six (RO-6; representing outermost layers 21–24), as a histogram (right panel). Data are represented as the mean ± SEM, *n* ≥ 5. Statistical significance (* *p* ≤ 0.05, ** *p* ≤ 0.01; **** *p* ≤ 0.0001) was assessed using a Student’s unpaired *t*-test; “ns”, non-significant.

The 3D-micrographs, obtained through super-resolution microscopy of MCF10A and MDA-MB-231 nuclei labeled with SUN2 (Figure 2A,B), underwent image processing. Specifically, the distribution of SUN2 labelling in the nuclear compartment was analysed by performing bioimage pre-processing and segmentation, to calculate the average SUN2 pixel intensity of 24 evenly-spaced 3D-regions across the 3D nuclei. Bioimage pre-processing: A widely adopted processing technique, namely Gaussian filtering, was employed to mitigate the effects of noise [46]. Gaussian smoothing, a method that leverages the Gaussian distribution to compute a weighted average of neighbouring voxels, was employed to achieve noise reduction and image smoothing. This technique operates as a convolution-based filter, using a Gaussian kernel as its basis. Bioimage segmentation: This step is pivotal, dividing an image based on shared characteristics, such as colour or texture. We employed Otsu’s thresholding method [47] to segment nuclei (foreground) from the background, automatically determining an optimal threshold. Pixels above the threshold are labelled as foreground (white), while those with lower intensity or equal to the threshold are designated as background (black), which yields a binary image. Otsu’s method efficiently separates classes. Following segmentation, morphological operations [48] are applied to refine the object of interest. These operations, which include dilation, erosion, and closing, employ a structuring element (kernel) to modify the image based on the local pixel values. Boundary object elimination: To eliminate objects touching the image border, that might not be relevant to the analysis, an extra step is required, particularly for 3D datasets. This step focuses on the extreme *X* − *Y* planes, ensuring that only objects on the edges of the entire volume are removed. This prevents discarding actual data within the volume by mistake, particularly the first and last slices along the Z-axis. Distance transform analysis of SUN2 protein spread: To quantify the spatial distribution of SUN2 within the nucleus, we employed distance transform [49], a well-established image processing technique with various applications [50]. In our analysis, it is applied to a binary image (*I_seg*), where foreground pixels denote the nucleus and the background pixels represent the cell interior. The DT algorithm produces a distance map (*I_dt*) of identical dimensions to *I_seg*. Each voxel in *I_dt* contains a value representing the shortest distance from that voxel to the nearest boundary voxel in *I_seg*. Distance transform was then used to define 24 nuclear 3D-layers, within which the average pixel intensity was calculated. 

The entire software was written in Python. 

### 2.10. Statistical Analysis

The analysis was conducted using different statistical methods, based on the number of groups being analysed. Where more than two groups were analysed, a one-way ANOVA (analysis of variance), followed by a Dunnett’s post-hoc test, was employed to determine the significance compared to the control (vehicle). This analysis was carried out using Graphpad Prism version 10.1.1 (GraphPad, San Diego, CA, USA). When analysing two groups, a Student’s unpaired *t*-test was conducted using Microsoft Excel version 16.66.1. The data were presented as mean ± SEM (standard error of the mean), with significance determined at a *p*-value of ≤ 0.05.

## 3. Results

### 3.1. Disulphide Bond-Mediated Oligomerisation of SUN2 and LAMIN B1 Is Prominent in MCF10A Cells, but Not in MDA-MB-231 Cells

By employing a standard Western blot analysis, the expression levels for SUN1 and SUN2 relative to GAPDH were assessed in both MCF10A and MDA-MB-231 cells (Figure 1A). Quantitative evaluation of the Western blot data indicated significant changes in the SUN1 and SUN2 expression profiles. In contrast to the observed SUN1 overexpression (2.13-fold change), SUN2 protein levels were significantly downregulated (0.52-fold change) in MDA-MB-231 cells compared to the control breast epithelium cell line MCF10A (Figure 1B). Next, the subcellular localisation of both SUN1 and SUN2 proteins was examined using standard confocal immunofluorescence microscopy. Both SUN-domain proteins were found exclusively at the NE in MCF10A, and no obvious differences for the staining intensity and protein distribution were observed when compared to MDA-MB-231 cells (Figure 1C). Earlier findings in our laboratory suggested that denaturation at high temperatures (>70 °C) influences the accurate determination of SUN1 levels using Western blotting, due to the formation of protein aggregates that cannot be resolved using SDS-PAGE [21]. Considering the involvement of disulphide bridges in tertiary and quaternary SUN-domain structures [21,23], the decision was made to conduct further Western blotting analysis under both reducing (Figure 1D) and non-reducing (Figure 1E) conditions, with protein samples incubated at 50 °C before SDS-PAGE. In addition, the utilisation of gradient gels helped the characterisation of higher-order protein complexes. Immunoblotting against SUN1 detected a major SUN1 band >100 kDa (SUN1: ENST00000405266.5; predicted molecular weight 91.1 kDa), and a weaker signal at 95 kDa, in both MCF10A and MDA-MB-231 cells (Figure 1D). For Lamin B1, the anticipated 72 kDa protein was detected in both cell types and, strikingly, a weaker yet consistent signal at 95 kDa was observed exclusively in MCF10A cells (Figure 1D). Moreover, the SUN2 Western blot experiments detected a major ~80.0 kDa band (highlighted by a green asterisk), closely reflecting the theoretical SUN2 molecular weight (SUN2: ENST00000405018.5; 82.5 kDa), as well as an additional, less prominent band at 72 kDa, in both cell lines (Figure 1D). However, in agreement with Figure 1B, quantitative analysis of the SUN2 (82.5 kDa) expression revealed a significant reduction (0.6-fold) in MDA-MB-231 cells relative to the control (Figure 1D). Next, the contribution of disulphide bridges to the possible oligomerisation of SUN1, SUN2, Lamin A/C, and Lamin B1 was evaluated by conducting Western blotting on MCF10A and MDA-MB-231 non-reduced protein cell lysates. This experiment revealed multiple SUN1 and SUN2 bands and, while complex, a distinctive profile for the SUN1 protein was evident. Although SUN1 monomers were clearly detectable in both cell types examined, MDA-MB-231 cells showed a dominance for large >200 kDa oligomers, whereas 130–200 kDa SUN1 complexes dominated in MCF10A cells. Compared to SUN1, the SUN2 protein pattern was less complex, with only two major bands being detectable in MCF10A cells. The SUN2 monomer band (~80 kDa) was clearly detectable (Figure 1E; green asterisk) and accompanied by a second equally strong signal, appearing at >140 kDa (Figure 1E; blue asterisk). In contrast, only the SUN2 monomer protein was detectable in MDA-MB-231 cells. Quantitative analysis of the high molecular weight SUN2 signal (>140 kDa) relative to the SUN2 monomer (~80 kDa) pool, suggests a ~1:1 ratio in MCF10A cells. When the total SUN2 content (monomer and >140 kDa protein pools combined) in MCF10A cells is compared to the SUN2 levels in MDA-MB-231 cells, a 0.5-fold reduction in SUN2 expression is observed in the latter cell line. In addition, the content of the SUN2 monomer in MCF10A and MDA-MB-231 is similar, with fold-expression values of approximately 0.5033 and 0.5040, respectively. Altogether, the above evidence suggests that the high molecular weight of SUN2 signal detected in MCF10A non-reducing cell lysates corresponds to a SUN2 homodimer. The examination of Lamin A/C protein did not reveal any other additional molecular arrangements. In sharp contrast, Lamin B1 Western blotting revealed two distinct signals in MCF10A cells. The first band corresponded to a 72 kDa protein, indicative of a Lamin B1 monomeric protein. The second signal, however, indicated a 140 kDa protein, suggesting a Lamin B1 homodimer, which was not detected in MDA-MB-231 lysates. In summary, the data indicate stark differences in the disulphide bond-mediated oligomerisation of Lamin B1, SUN1, and SUN2 between MCF10A and MDA-MB-231 cells. 

### 3.2. Super-Resolution Imaging Suggests the Presence of Higher-Order Molecular Structures for SUN2 at the Periphery of the MCF10A Nucleus, Which Are Absent in MDA-MB-231 Cells

As the previous experiments were conducted under SDS solubilisation conditions (i.e., SDS-PAGE), we were intrigued by the observed changes in the disulphide cross-linking of SUN2 in MCF10A and MDA-MB-231 cells. Consequently, our next aim was to document and corroborate whether these alterations impacted the subcellular distribution of SUN2 using high-resolution imaging (XY resolution ~ 140 nm; Z-resolution ~ 400 nm). To reduce pre-existing disulphide bonds in proteins, cells were treated with 1 μM DTT (Dithiothreitol) for 30 min prior to fixation, and compared with untreated cells before super-resolution imaging to generate 3D-images (Figure 2A,B). Maximum intensity XY projections of both untreated and DTT-treated MCF10A cells revealed a homogenous dotty pattern for SUN2 across the entire ovoid-shaped nucleus. Sagittal and transverse views of the SUN2 stain also unveiled a disc-like and flat morphology for their nuclei (Figure 2A). In contrast, MDA-MB-231 nuclei (Figure 2B) appeared taller (XZ and YZ projections) with an irregular morphology (denoted by yellow arrowheads). Importantly, the SUN2 immunolabelling was uneven, suggesting the presence of protein aggregates and prominent nuclear folds (Figure 2B, white arrowheads). To gain further insights into the distribution of SUN2, 3D-micrographs of SUN2-labelling underwent image processing (Figure 2C; for more details we refer the reader to the M and M section). Specifically, the respective nuclei were divided into 24 concentric and equally-spaced 3D shells, and the mean pixel intensity of SUN2 was quantified within each 3D layer. A graphical representation of the deduced values for the two examined cell types indicates an asymmetric SUN2 staining distribution (Figure 2D). The mean SUN2 pixel intensity is the lowest in the nuclear centroid (layer 1), which progressively increases, reaching a plateau between layers 12–24 within the MCF10A nuclei. An asymmetric distribution was also observable for MDA-MB-231 nuclei. However, the exhibited values for SUN2 were significantly higher and non-uniformly distributed towards the edge of the nucleus (post layer 12) when compared to the control MCF10A cells. Specifically, when comparing the mean SUN2 pixel intensity values for the innermost layers 1–4 (region of interest-1 [RO-1]) to the outermost layers 20–24 (region of interest-6 [RO-6]), a significant ≥1.97-fold increase was observed for the latter layers in both MCF10A and MDA-MB-231 cells (see Supplement Figure S1A). Interestingly, the radial gradient of SUN2 pixel intensity from the nuclear centre (RO-1) to the periphery (RO-6) remained intact in both MCF10A (See Supplement Figure S1B) and MDA-MB-231 cells (Figure S1C), even after DTT treatment. Furthermore, DTT treatment exerted significant changes for the outermost RO (RO-6), but not in RO-5 in MCF10A nuclei (Figure 2E), when compared to the respective control, while no significant changes were observed in DTT-treated versus untreated MDA-MB-231 nuclei (Figure 2F). In summary, the super-resolution image analysis revealed some similarities, but also some substantial differences, with respect to SUN2 organisation between MCF10A and MDA-MB-231 nuclei. In particular, the imaging analysis revealed roles for disulphide bond-mediated interchain SUN2 interactions in a specific sub-nuclear region in MCF10A cells (i.e., nuclear periphery; RO-6), but not in MDA-MB-231 cells.

### 3.3. PDI inhibitors, 16F16 and PACMA31, Upregulate HO-1 Protein Levels and Modulate the Expression of Core LINC Complex Proteins and Associated Nuclear Lamina Proteins in MDA-MB-231 Cells

Due to the well-known roles of members of the PDI family in disulphide bond dynamics/homeostasis, the levels of PDIA1 protein were examined in MCF10A and MDA-MB-231 cell lysates using Western blotting (Figure S2A). No significant changes in PDIA1 expression were observed between the two cell lines (Figure S2B). Subsequently, immunofluorescence analyses of PDIA1 and Nesprin-2 were conducted to assess potential differences in their subcellular organisation (Figure S2C). No obvious differences were observed between the PDIA1 localisation in MCF10A and MDA-MB-231 cells. The majority of the PDIA1 protein was found in the cytoplasmic compartment, displaying a punctate pattern, with some occasional labelling of the NE, partially co-localising with Nesprin-2. Nesprin-2 itself exclusively localised to the NE of MCF10A cells, whereas in MDA-MB-231 cells, a substantial signal for Nesprin-2 was found in the cytoplasm (Figure S2C; arrowheads). 

**Figure 3:**
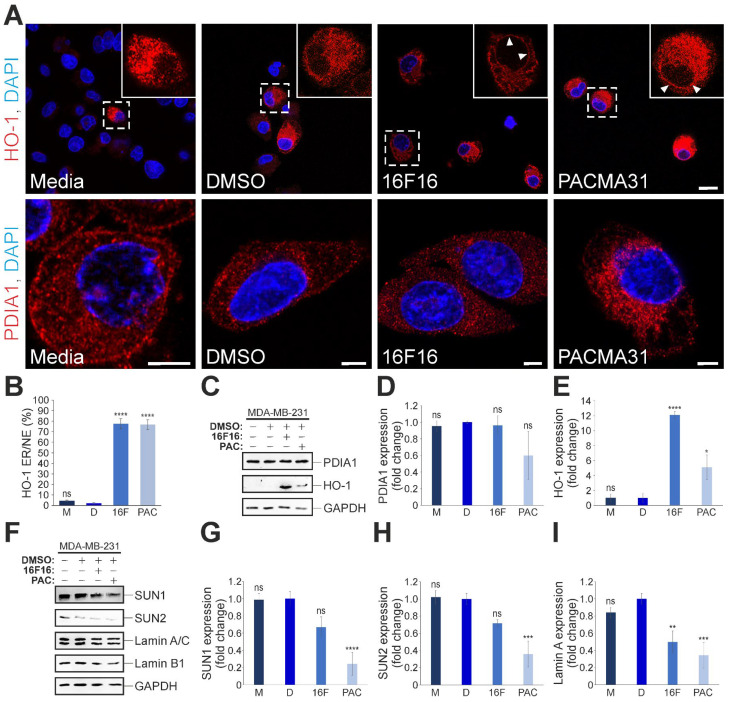
PDI inhibition affects the expression of HO-1, SUN1, SUN2 and Lamin A/C proteins in MDA-MB-231 cells. (**A**) Representative confocal microscopy images of HO-1 (top row) and PDIA1 (lower row) immunostained MDA-MB-231 cells treated with media only, DMSO (vehicle), 5 μM 16F16, and 2.5 μM PACMA31. Insets (only the red channel is displayed) represent higher magnifications of areas indicated by dashed lines. Arrowheads mark the ER/NE staining of HO-1. Nuclei are visualised by DAPI stain. Scale bar top row: 20 μm; scale bar lower row: 5 μm. (**B**) Quantification of the percentage (%) of cells that exhibit prominent ER/NE HO-1 localisation for MDA-MB-231 cells incubated with media only (M), DMSO (vehicle; D), 5 μM 16F16 (16F) and 2.5 μM PACMA31 (PAC); >150 cells analysed per condition. (**C**) Immunoblot analysis of PDIA1 and HO-1 expression in PDI-inhibited (16F, PAC) and control MDA-MB-231 cells, normalised against GAPDH protein levels, which indicates equal loading of protein. (**D**) Quantification of PDIA1 protein expression in PDI-inhibited (16F, PAC) versus untreated (M, D) MDA-MB-231 cells. (**E**) Quantification of HO-1 protein levels in untreated controls (M, D) versus PDI-inhibited (16F, PAC) MDA-MB-231 cells. (**F**) Representative Western blot analysis of SUN1, SUN2, Lamin A/C, Lamin B1, and GAPDH expression in untreated versus PDI-inhibited cells. (**G**–**I**) Quantification of SUN1 (**G**), SUN2 (**H**), and Lamin A/C (**I**) protein expression levels. The data in graphs (**B**,**D**,**E**,**G**–**I**) are represented as the mean ± SEM, with a sample size of *n* = 3. Statistical significance was conducted using one-way ANOVA with a Dunnett’s post-hoc test relative to the baseline DMSO (**D**) control treatment. Lack of significance is denoted as “ns”, whereas the asterisk (*) indicates *p* ≤ 0.05, ** *p* ≤ 0.01, *** *p* ≤ 0.001, **** *p* ≤ 0.0001.

To gain insights into the broader involvement of PDI-family members in LINC complex biology, we next assessed the consequences of PDI inhibition in MDA-MB-231 cells. To determine a suitable concentration for the PDI inhibitors 16F16 and PACMA31, an end point toxicity assay, in the form of an MTT, was carried out. This assay measures cell proliferation and cell viability, as well as metabolic activity. Therefore, the MTT was carried out in serum-free media (no FBS, to restrict proliferation), as well as in media containing FBS (complete media). Our results indicate that PACMA31 affects the cell proliferation, viability and metabolic activity of MDA-MB-231 cells (Figure S3). In complete media, it can be inferred that PACMA31 is strongly cytotoxic within the concentration range used (0.5–10 μM PACMA31), resulting in a decrease in live cells of ~80%. However, in serum-free media, a reduction in live cells of >50% is restricted to 5 and 10 μM PACMA31 concentrations, with ~80% cell survival observed in cells receiving the lower PACMA31 concentrations (e.g., 0.5, 1, and 2.5 μM) (Figure S3). Therefore, a 2.5 μM concentration for PACMA31 was chosen for subsequent experiments. In contrast, with 16F16 treatment, no clear differences were observed between complete and serum-free media conditions (Figure S3). Therefore, the changes observed are most likely due to the cytotoxicity of the drug, with limited effects seen at the two higher concentrations (cell viability: ~60% for 10 μM and ~80% for 5 μM 16F16). Hence, a concentration of 5 μM for 16F16 was chosen. To evaluate if the chosen PDI inhibitor concentrations elicited stress responses in the treated cells, immunofluorescence staining was performed against Heme oxygenase-1 (HO-1). HO-1 is a microsomal enzyme that plays important roles in protecting cells against oxidative stress. In untreated (media) and vehicle (DMSO)-treated cells, a faint stain for HO-1 was observed sporadically. In contrast, the majority of 16F16 and PACMA31-treated cells strongly expressed HO-1 (Figure 3A; top row). Upon closer examination, the subcellular localisation of HO-1 revealed a prominent reticular pattern in the cytoplasm (endoplasmic reticulum [ER]) and a pronounced staining of the NE (arrowheads in Figure 3A). Subsequently, the percentage of cells that exhibited strong HO-1 labelling in either the ER and/or the NE (ER/NE) of the examined cells was measured. The graphical display of the results (Figure 3B) shows that 77.6% of 16F16-treated and 76.6% of PACMA31-treated cells display strong HO-1 staining at the ER/NE, compared to 4.6% (media) and 2.1% (DMSO) of control cells. In comparison to HO-1, no obvious effects were seen for the PDIA1 protein sub-cellular distribution and the PDIA1 staining intensity in both treated and untreated control cells (Figure 3A, lower row). Next, the consequences of PDI inhibition on HO-1, PDIA1, SUN-domain, and nuclear lamina protein expression were evaluated by Western blotting. Equal loading of cell lysates was corroborated by staining the gels after SDS-PAGE with InstantBlue Coomassie (Figure S4) and immunoblotting against the housekeeping protein, GAPDH. Consistent with the immunofluorescence data, no significant changes in PDIA1 protein expression were observed in PDI-inhibited cells (Figure 3C,D). In stark contrast to PDIA1, the expression of HO-1 increased 12-fold and 5-fold, compared to the corresponding control (DMSO [D]; vehicle), in 16F16 and PACMA31-treated cells, respectively (Figure 3E). 

While SUN1 and SUN2 expression decreased with 16F16 treatment (Figure 3F–H), the changes were not statistically significant. Compared to cells exposed to 16F16, PACMA31 treatment had a more pronounced effect on protein expression of both SUN1 and SUN2. Specifically, a 0.24-fold reduction in SUN1 and a 0.36-fold reduction in SUN2 were observed, compared to the vehicle (Figure 3G,H). Moreover, pharmacological PDI inhibition by both 16F16 and PACMA31 significantly reduced the expression of Lamin A protein. In detail, a 0.5-fold reduction and a 0.34-fold reduction in Lamin A protein levels were observed in MDA-MB-231 cells treated with 16F16 and PACMA31, respectively, relative to the control (Figure 3I). PDI inhibition also resulted in reduced Lamin B1 expression. However, the changes were only statistically significant for the PACMA31 treatment (i.e., 0.22-fold reduction; Figure S5). In summary, the results indicate that upon PDI inhibition, the levels of SUN-domain and A-type and B1-type lamin proteins are reduced. 

### 3.4. Pharmacological Inhibition of PDIs by 16F16 and PACMA31 Displaces Nesprin-2 from the Nuclear Envelope and Alters Perinuclear F-actin Architecture in MDA-MB-231 Cells

**Figure 4:**
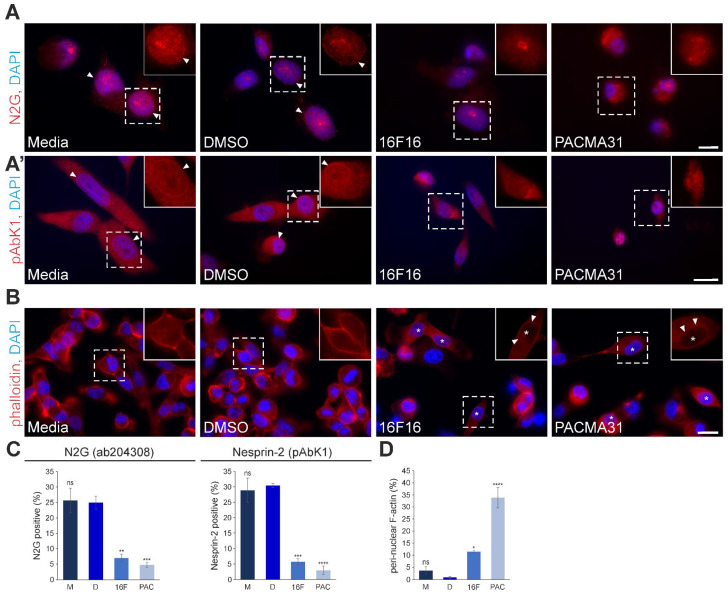
PDI inhibition disrupts the nuclear envelope localisation of Nesprin-2 isoforms, including Nesprin-2 giant, and results in the formation of perinuclear F-actin rings. (**A**,**A′**) Representative immunofluorescence microscopy images of MDA-MB-231 cells incubated with media, DMSO (vehicle), 5 μM 16F16, and 2.5 μM PACMA31, immunostained with Nesprin-2 giant (N2G) isoform-specific (Ab204308) and generic Nesprin-2 isoform (pAbK1) antibodies. DAPI denotes nuclear staining. Insets (only the red channel is displayed) are higher magnifications of dashed boxed areas. Arrowheads indicate the Nesprin-2 presence at the NE. Note the absence of Nesprin-2 staining in the nuclei of PDI-inhibited cells. Scale bar for panel **A**: 10 μm; scale bar for panel **A′**: 20 μm. (**B**) Fluorescence microscopy examination of TRITC-phalloidin (labels F-actin) and DAPI-stained MDA-MB-231 control cells relative to PDI-inhibited cells. Note that the localisation of F-actin in control cells is mainly cortical. Upon PDI inhibition a substantial number of cells (asterisks) exhibit F-actin staining around the NE (arrowheads). Insets (only the red channel is displayed) are higher magnifications of the regions delineated by dashed lines. Scale bar: 20 μm. (**C**) Quantitative examination of the Nesprin-2 Ab204308 antibody (detects Nesprin-2 giant; N2G) and pAbK1 antibody NE staining pattern in control (Media [M], vehicle [D]) and PDI-inhibited (16F16 [16F], PACMA31 [PAC]) cells, with more than 300 cells examined for each condition. (**D**) Quantification of the percentage (%) of cells exhibiting perinuclear F-actin rings under control (M, D), and PDI-inhibited (16F, PAC) conditions is indicated. Over 400 cells were analysed for each specific condition. The data in graphs (**C**,**D**) are represented as the mean ± SEM, with a sample of *n* = 3. Statistical significance was determined using one-way ANOVA with a Dunnett’s post-hoc test, comparing to the vehicle treatment (DMSO; [D]). Non-significant differences are denoted as “ns”, * *p* ≤ 0.05, ** *p* ≤ 0.01, *** *p* ≤ 0.001, **** *p* ≤ 0.0001.

Given the established importance of SUN1, SUN2, and nuclear lamina proteins in facilitating the proper recruitment of nesprins to the NE, a more detailed exploration of nesprin localisation to the NE was conducted using epifluorescence microscopy. Based on previously published work, it was decided to focus exclusively on *SYNE2*-encoded isoforms (i.e., Nesprin-2), which are broadly expressed in mammalian tissues and strongly expressed in epithelia. In addition, to establish a comprehensive understanding, multiple Nesprin-2 antibodies were employed. For a more focused understanding, Ab204308 was utilised to detect the largest *SYNE2*-encoded isoform, Nesprin-2 giant, which is an actin-binding domain (ABD)-containing protein with a molecular weight exceeding 800 kDa. The general Nesprin-2 antibody pAbK1 was also used [11], as this detects Nesprin-2 giant as well as smaller KASH-domain-containing isoforms. In untreated (media) and vehicle-treated (DMSO) cells, prominent Nesprin-2 giant staining was observed at the NE (arrowheads in Figure 4A and insets). In sharp contrast, the localisation of Nesprin-2 giant to the nucleus was compromised in PDI-inhibited cells (Figure 4A). As anticipated, Nesprin-2 pAbK1 detected additional cellular structures, including the NE (Figure 4A’ arrowheads) and the cytoplasmic compartment in control cells (Figure 4A’). Despite this, similarly to Nesprin-2 giant, we observed a reduction of pAbK1 NE-labeling in cells subjected to 16F16 and PACMA31 treatment (Figure 4A’). Quantitative analysis of the Nesprin-2 antibody staining revealed that PDI inhibition induced significant effects on the NE localisation of the protein. Compared to untreated cells (25.7%) and vehicle-treated (DMSO; 24.9%) cells, only 7% of cells exposed to 16F16 and 4.8% of PACMA31-treated cells displayed robust Nesprin-2 giant NE staining (Figure 4C). Furthermore, the proportion of cells exhibiting pronounced pAbK1 positive staining at the nucleus was notably lower in PDI-inhibited cells; 5.7% of 16F16 exposed cells and 3% of PACMA31-treated cells, compared to 28.8% of untreated cells (media) and 30.4% in vehicle-treated (DMSO) cells. As Nesprin-2 giant is an F-actin associated protein, the effects on F-actin architecture were then examined in untreated and PDI-inhibited cells using fluorescently-coupled phalloidin, which reveals filamentous actin structures (Figure 4B and Figure S6). In control cells, most F-actin staining is found at the cell cortex, with the majority of nuclei being devoid of prominent F-actin structures. In sharp contrast, a substantial fraction of PDI-inhibited cells (highlighted with asterisks) displayed pronounced ring-like and F-actin positive structures at the nucleus (arrowheads in Figure 4B and Figure S6). Statistical evaluation of this immunofluorescence data revealed a significant increase in perinuclear F-actin structures in 16F16 exposed (11.4%) and PACMA31-treated (33.8%) cells (Figure 4D), relative to controls. In control cells, the presence of F-actin around the nucleus was infrequent, with only 3.7% of untreated (media) and 0.9% of vehicle-treated cells showing weak perinuclear F-actin rings. Collectively, the data indicate that PDI inhibition impedes the recruitment of Nesprin-2 isoforms to the NE and redistributes F-actin towards the nucleus. 

### 3.5. Differential Effects of 16F16 and PACMA31 Treatment on the Expression and Organisation of Cytoplasmic Intermediate Filaments in MDA-MB-231 Cells

**Figure 5:**
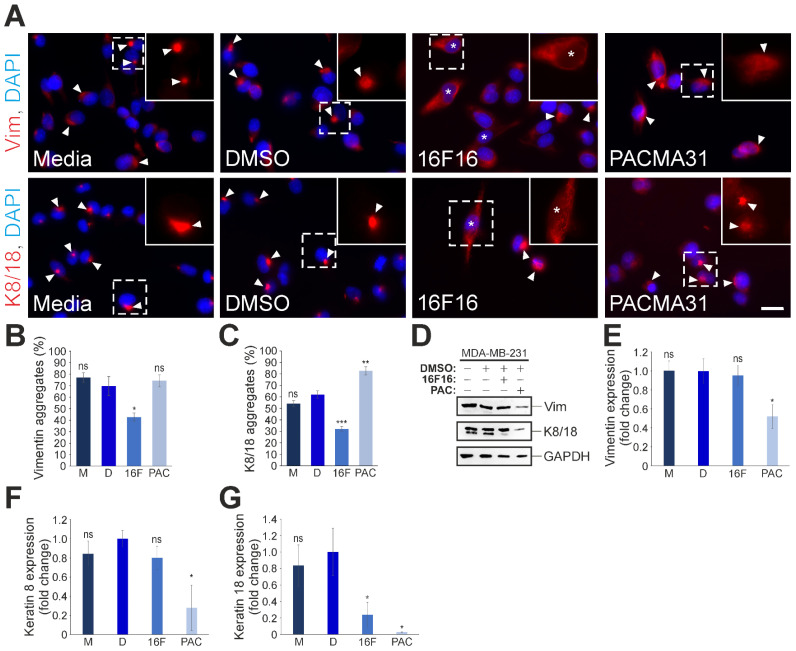
PDI inhibition affects the Vimentin and Keratin 8/18 intermediate filament cytoskeleton in MDA-MB-231 cells. (**A**) Exemplary immunofluorescence microscopy images of the Vimentin (Vim) and Keratin 8/18 (K8/18) cytoskeletal organisation in control (media [M] and DMSO [D; vehicle]) and 5 μM 16F16 (16F) and 2.5 μM PACMA31 (PAC)-treated cells. The arrowheads indicate the existence of perinuclear cytoskeletal aggregations. Insets (only the red channel is displayed) are higher magnifications of the dashed boxed areas. Asterisks denote cells with a well-dispersed IF cytoskeleton devoid of aggregates. Nuclei are visualised by DAPI staining. Scale bar: 20 μm. (**B**) The percentage (%) of cells displaying perinuclear Vimentin aggregates is depicted in both untreated (M), DMSO (D; vehicle), and drug-treated (16F and PAC) cells. Over 300 cells were examined per condition. (**C**) Shows the percentage (%) of cells exhibiting perinuclear K8/18 aggregates in both untreated and drug-treated cells. More than 300 cells were examined per specific condition. (**D**) Western blot analysis of Vimentin and K8/18 protein expression levels in control and PDI-inhibited cells. Equal protein loading was confirmed using antibodies against GAPDH. (**E**–**G**) Comparative quantification of Vimentin (**E**), Keratin 8 (**F**), and Keratin 18 (**G**) protein expression levels (fold change) in untreated (M) and drug-treated cells in relation to the normalised vehicle (**D**) control sample.The data in graphs (**B**,**C**,**E**–**G**) are represented as the mean ± SEM, with a sample of n = 3. Statistical significance was determined using one-way ANOVA with a Dunnett’s post-hoc test, compared to the vehicle treatment (DMSO; [D]). Non-significant differences are denoted as “ns”, * *p* ≤ 0.05, ** *p* ≤ 0.01, *** *p* ≤ 0.001.

Building upon the differences in nuclear IFs and the F-actin cytoskeleton following PDI inhibition, an extension of the study was pursued to include immunofluorescence examination of other cytoskeletal filaments, namely Vimentin (Vim) and Keratins 8/18 (K8/18). For both Vimentin and K8/18, control cells exhibited pronounced and highly-localised perinuclear accumulation (arrowheads in Figure 5A), which persisted in PACMA31-exposed cells. Whereas, following 16F16 treatment, cells were noted to display well-spread Vimentin and K8/18 networks (denoted by asterisks in Figure 5A). To acquire a more profound understanding of the protein accumulation characteristics, a quantitative assessment of Vimentin and K8/18 cytoskeletons was conducted. Compared to untreated (77%), vehicle-treated (69.6%), and PACMA31-exposed (74.2%) MDA-MB-231 cells, 16F16 treated cells showed a significant reduction in Vimentin accumulation (42.6%) (Figure 5B). Regarding K8/18, aggregates were present in 54.2% of untreated and 62% of vehicle (DMSO; D)-exposed cells (Figure 5C). However, after pharmacological intervention with 16F16, K8/18 aggregation was significantly reduced to 32.1%. Conversely, PACMA31 exposure led to an increase in K8/18 aggregation, with 82.6% of cells displaying this phenotype. Next, the protein levels for Vimentin and K8/18 were examined in control and PDI-inhibited cells using Western blotting (Figure 5D). As indicated in Figure 5E, PACMA31 treatment significantly reduced Vimentin expression (0.5-fold), whereas 16F16 treatment had no significant effects (0.95-fold change). Similarly, Keratin 8 expression was significantly reduced in PACMA31-treated cells (0.28-fold change), but not following 16F16 exposure (0.8-fold change, Figure 5F). In contrast, Keratin 18 protein levels were significantly reduced by both 16F16 (0.23-fold change) and PACMA31 (0.02-fold change) (Figure 5G). Finally, Western blotting for cytoskeleton proteins using pan-keratin, β-tubulin, and β-actin antibodies, revealed significant reductions in their levels in PACMA31-treated cells only (Figure S7). To summarise, we found that both Vimentin and Keratin 8/18 accumulated near the nucleus in the triple-negative breast cancer cells. This phenotype was “rescued” to a more normal network by the PDI inhibitor 16F16, but not by PACMA31. Surprisingly, PACMA31 did reduce Vimentin and Keratin 8 expression, but 16F16 did not. However, Keratin 18 expression was reduced by both inhibitors. In summary, this highlights that 16F16 and PACMA31 treatments induce distinct molecular changes and cellular phenotypes.

### 3.6. Inhibition of PDIs by 16F16 and PACMA31 Reduces Cell Proliferation, Cell Motility, and Polarity in 2D Scratch Wound Assays, While Facilitating the Migration of MDA-MB-231 Cells in Space Restrictive in Vitro Environments

**Figure 6:**
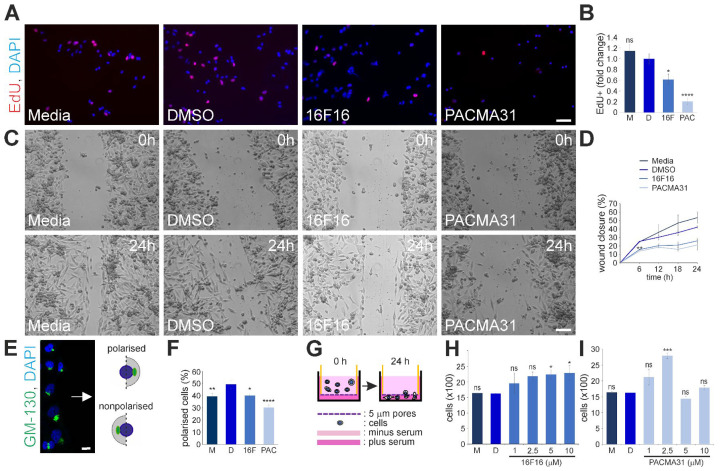
Pharmacological inhibition of PDI activity in MDA-MB-231 cells reduces cell proliferation, 2D cell migration, and cell polarity, while promoting their migration through space-restrictive 5 μm porous surfaces. **(A)** Confocal microscopy micrographs of EdU proliferation assay results depict untreated, vehicle-treated (DMSO), and drug-treated (i.e., 5 μM 16F16 and 2.5 μM PACMA31) MDA-MB-231 cells. DAPI staining denotes nuclei. Scale bar: 50 μm. (**B**) Comparative quantification of EdU incorporation (fold change) in untreated (M) and drug-treated (5 μM 16F16 [16F] and 2.5 μM PACMA31 [PAC]) cells in relation to the normalised vehicle (D) controls. (**C**) Phase contrast images of cell wounding experiments at 0 h and 24 h post-wounding, involving untreated and PDI-inhibited cells. Scale bar: 100 μm. (**D**) Graph illustrating the percentage (%) of wound closure at 0 h, 6 h, 12 h, and 24 h time points. (**E**) Confocal microscopy image of the free wound edge of a MDA-MB-231 2D monolayer stained for the Golgi apparatus (GM-130) and the nucleus (DAPI). The direction of cell movement is indicated with the white arrow. Scale bar: 10 μm. The schematic on the right illustrates the criteria employed to distinguish between polarised and non-polarised cells. Cells were considered polarised when the Golgi organelle (depicted in green) was oriented within a 180-degree radius (depicted in grey) facing the free wound edge. The nucleus is depicted as a blue circle. (**F**) The graph illustrates the percentage (%) of polarised cells for the specified drug-treatments relative to the control conditions. (**G**) A schematic representation of cell migration is shown, employing inserts containing a space-restrictive porous (Diameter: 5 μm) membrane (dashed purple line) and serum-based attractants. The light pink shading indicates the lack of serum, while the dark pink shading denotes the presence of serum. The schematic captures cell behaviour at the 0 h (0 h) seeding and 24 h (24 h) post-seeding time points. (**H**) Graph indicating the absolute numbers of cells that migrated to the basal side of the membranous porous insert under the specified concentrations of the 16F16 drug, in comparison to control conditions. (**I**) The presented data illustrate the absolute numbers of cells that migrated to the basal side of the porous membrane insert under the defined concentrations of the PACMA31 drug, relative to control conditions. The data in graphs (**B**,**D**,**F**,**H**,**I**) are represented as the mean ± SEM, with a sample of n = 3. Statistical significance was determined using one-way ANOVA with a Dunnett’s post-hoc test, compared to the vehicle treatment (DMSO; [D]). Non-significant differences are denoted as “ns”, * *p* ≤ 0.05, ** *p* ≤ 0.01, *** *p* ≤ 0.001, **** *p* ≤ 0.0001.

To gain comprehensive insights into the cell biology of PDI-inhibited cells, further analysis was conducted. An EdU assay revealed that 16F16 and PACMA31 treatment significantly reduced cell proliferation (Figure 6A,B). Given the effects observed on both F-actin and IF networks, we then determined whether this would affect cell movement and cell polarity. Wound closure was delayed in PDI-inhibited cells, with significant changes observed at the 6-h post-wound time point (Figure 6C,D and Figure S8). Examination of Golgi apparatus polarisation towards the cell-free wound edge (Figure 6E) revealed significant defects in cells treated with PDI inhibitors (Figure 6F). The utilisation of space-restrictive porous inserts (pore diameter: 5 μm; Figure 6G) revealed that 5 μM and 10 μM 16F16, as well as 2.5 μM PACMA31, significantly enhanced the migratory capacity of MDA-MB-231 cells (Figure 6H,I). However, no effects were observed at concentrations of 5 and 10 μM for PACMA31.

## 4. Discussion

This study demonstrates that PDI family members regulate LINC complex stability at the NE, affecting cell migration and invasiveness. Moreover, the data indicate oligomerisation differences in SUN2 and Lamin B1, between non-invasive mammary epithelium (MCF10A) and invasive cancer (MDA-MB-231) cells in vitro, suggesting a mechanism by which the LINC complex influences breast cancer cell behaviour.

**Figure 7:**
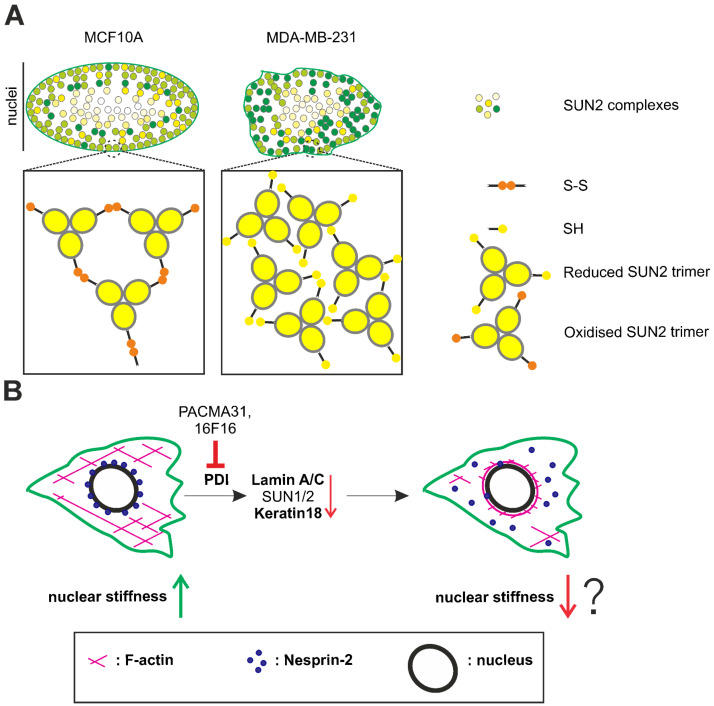
A schematic depiction of SUN2 macromolecular organisation in the nuclei of untreated MCF10A and MDA-MB-231 cells (**A**), along with the consequences of PDI inhibition on LINC complex composition and perinuclear F-actin structure (**B**). (**A**) Nuclear 2D projection (XY plane). SUN2 macromolecular assemblies are depicted as small coloured circles. The colours create a gradient with varying shades from “light yellow” to “dark green”, aiming to reflect changes in pixel intensity, transitioning from lower to higher values. Note the prevalence of “green” SUN2 structures at the periphery and “light yellow” SUN2 complexes in the centre; aiming to reflect that in MCF10A nuclei, SUN2 staining pixel intensity is at the highest at the periphery and lowest in the centre. The distribution of SUN2 complexes is even throughout the entire nucleus. In MDA-MB-231 cells, the gradient of SUN2 pixel intensity from the periphery to the centre is maintained, yet the organisation of SUN2 complexes is less orderly. The insets below aim to offer a more detailed view at the molecular level. For simplicity, SUN2 is depicted as a trimer, despite ample evidence that other molecular assemblies exist. The SUN2 cysteines are depicted as small “lollipops”. Reduced cysteines (SH) are illustrated in yellow, while oxidised cysteines (S–S) are portrayed in orange. Within MCF10A nuclei, oxidised SUN2 trimers are present, displaying a preference for localisation at the nuclear periphery. This localisation could potentially facilitate uniform SUN2 complex spacing and limit the overcrowding of SUN2 molecules. In stark contrast, MDA-MB-231 cells exhibit SUN2 trimers lacking stabilisation and equal spacing through disulphide bonds, resulting in SUN2 aggregation, as indicated by the “dark green” colouration of SUN2 complexes. (**B**) The primary consequences on an MDA-MB-231 cell following PDI inhibition are summarised, with a specific focus on the LINC complex and F-actin architecture. Protein expression effects common to both PACMA31 and 16F16 treatments are shown in bold (i.e., Lamin A/C and Keratin 18 downregulation). Upon PDI inhibition, the expression of anchoring proteins for Nesprin-2, such as Lamin A/C and SUN-domain proteins, are reduced. This potentially leads to the untethering of Nesprin-2 from the outer nuclear membrane. The model suggests that the combination of these molecular events results in decreased nuclear stiffness, which facilitates cellular migration through space restrictive scaffolds in vitro. Moreover, it is hypothesised that the effects on nuclear pliability and structure are counteracted by the development of an F-actin perinuclear cage in drug-treated cells.

Firstly, using non-reducing SDS-PAGE, disulphide bond-mediated SUN2 and Lamin B1 homodimers were evident in MCF10A cells, which were not detectable in MDA-MB-231 cells. While the SUN1 pattern was also distinct, the complex banding pattern made interpretation more difficult, but we conclude that disulphide-linked oligomers were present in both cell types. Unlike SUN2, SUN1 possesses four cysteines within its C-terminus [21]. This likely contributes to a more intricate oligomerisation pattern [51], especially given the increased SUN1 expression in MDA-MB-231 cells compared to SUN2, which appears to have become disulphide-inert in these cells. Secondly, Supplementary Data involving super-resolution SUN2 imaging and bioimage analyses detected changes in the SUN2 pixel intensity at the periphery of MCF10A nuclei after reducing agent treatment, whereas no such alterations were observed in MDA-MB-231 nuclei. Furthermore, both cell types exhibited a SUN2 staining intensity gradient (Figure 2 and Figure 7). However, in MDA-MB-231 cells, the SUN2 staining appeared more irregular compared to MCF10A cells. Based on these observations, we propose the hypothesis that SUN2–SUN2 disulphide dimers distribute asymmetrically within the MCF10A nucleus, with enrichment observed in a broad band at the nuclear periphery. Interestingly, in typical 2D cultured cells, the forces exerted on the nucleus and the distribution of nuclear stiffness determinants, such as A-type lamins, are spatially heterogeneous. It has been reported that atomic force microscopy (AFM) micro-rheology of isolated single HeLa cell nuclei unveiled that the outer nuclear region is distinguished by its viscosity and reduced stiffness, possibly aiding mechano-sensing, while the inner nuclear core displays elasticity and higher stiffness [52]. Using drastically shortened ABD-containing Nesprin-2 tension sensors, it was discovered that the equatorial and apical planes of the nucleus experience the greatest forces [53]. Osmotic stress experiments conducted on 3T3 fibroblasts revealed that the greatest nuclear deformation occurred along the vertical axis, with lesser deformation observed along the substratum plane. Furthermore, focal enrichment of Lamin A in specific nuclear regions rendered them less deformable [54]. Thus, in mouse fibroblasts it is observed that there is an inverse relationship between Lamin A protein content and local nuclear pliability. LINC complex-mediated tension limits the detection of Lamin A/C on the basal side of adherent cells, using specific antibodies that recognise conformational epitopes [55]. In contrast to Lamin A/C, Lamin B1 lacks vertical polarisation properties [56]. Strikingly, we identified significant changes in the disulphide-mediated homo-dimerisation of Lamin B1 in MCF10A and MDA-MB-231 cells. Previous literature indicates that Lamin B1 harbors Cys317 within the coiled coil 2B segment and participates in disulphide bond homodimer formation [57]. Murine SUN1 and SUN2 interact with both Lamin A/C and Lamin B1 [1,3,58]. Lamin B1, however, is unable to tether SUN2 to the NE in the absence of Lamin A/C [59,60]. Whether Lamin B1 is key to the nuclear compartment spatial asymmetry seen for SUN2 in MCF10A cells warrants further exploration. Nevertheless, the enhanced SUN2 network interconnectivity adjacent to the nuclear periphery of MCF10A cells could serve as an adaptation to counterbalance the increased mechanical load experienced by the equatorial region of the nucleus. Similar forces may act on the MDA-MB-231 nuclei, but there are differences in the SUN2 staining asymmetry within the nuclear compartment. The lack of SUN2–SUN2 network connections (e.g., lack of interchain disulphide bonds), combined with the reduced levels of SUN2, will likely affect the structural integrity and mechanical responses of the MDA-MB-231 nucleus, as suggested by previous studies [61]. These SUN2 network deficits could lead to nuclear deformations, as evidenced by the nuclear folds (Figure 2B) and SUN2 regional clustering (Figure 7).

The human SUN2 (UniProt identifier: Q9UH99) protein segment that resides in the NE lumen contains three cysteines; Cys563, Cys601, and Cys705. The current in vitro structural data for the SUN2 C-terminus–KASH complex do not indicate any free cysteines [23]. In this complex, SUN2 Cys563 forms a bond with Nesprin-2 KASH Cys6821, while Cys601 and Cys705 bind to each other. Evidently, SUN2 does indeed form intermolecular disulphide bridges in MCF10A (as shown in this study) and in mouse C2C12 cells [62]. In C2C12 mouse cells, the redox state of SUN2 affected its protein turnover, oligomerisation, KASH-domain binding and, consequently, cytoskeletal architecture. Specifically, critical roles were assigned to mouse SUN2 Cys577 (equivalent to human SUN2 Cys563) in disulphide bond oligomerisation [62]. Our assumption, therefore, is that additional SUN-domain structures may exist in situ in cells containing free cysteines, enabling the formation of higher-order oligomers. Given the established impact of tensile forces on SUN2 structure using steered molecular-dynamics simulations, this possibility is indeed feasible [63]. 

Endoplasmic reticulum disulphide oxidase 1-α (ERO1-α) is highly overexpressed in triple-negative breast cancer, including the MDA-MB-231 cell line, and constitutes a marker of poor prognosis in breast cancer [64,65]. Therefore, this evidence indicates significant changes in the ER redox status of metastatic breast cancer cells. Clearly ER redox regulation, and the diverse functions of its client proteins, notably the PDIs, need to be carefully correlated with the SUN2 proteome to unravel the intricate regulatory mechanisms that govern the structure and functionality of the LINC complex. 

The molecular and cellular analysis of 16F16- and PACMA31-treated cells indicates some similarities, but also some stark differences, which was anticipated considering their differential PDI substrate protein specificity [42,43,44]. In general, the cellular effects of PACMA31 treatment were more severe compared to 16F16-mediated PDI inhibition. The significant upregulation of HO-1 in drug-treated cells compared to untreated cells suggests that both compounds induced ER stress. It is possible that the elevated levels of HO-1 expression in 16F16-treated cells compared to PACMA31-treated cells may offer a more effective cytoprotection against oxidative stress [66]. In this study, both PDI inhibitors demonstrated the ability to reduce the protein expression of Lamin A, SUN1, and SUN2, to varying degrees. Additionally, both drugs impeded the Nesprin-2 localisation to the nucleus. Overall, the molecular changes in PDI-inhibited cells suggest a disruption of the LINC complex, which is known to result in NE dilation in mammalian cells [1]. ER stress is characterised by the expansion or swelling of the ER lumen [67]. Given the direct physical connectivity between the ER and the NE, it is plausible that ER stress-induced molecular and structural changes could affect nuclear composition and function. In murine muscle cells, thapsigargin-induced ER stress reduces the levels of SUN2 [68]. Interestingly, Nesprin-1, Nesprin-2, and Nesprin-3, but not SUN-domain proteins, are TMX4 (thioredoxin-related transmembrane protein 4)-binding partners [69]. After inducing acute ER stress with cyclopiazonic acid (CPA) in fibroblasts, the reduction of KASH-domains by TMX4 reductase activity leads to disassembly of disulphide bonds between KASH and SUN proteins, consequently causing enlargement of the perinuclear space [69]. Our findings suggest that disruption of the ONM–INM bridge may also occur from nesprins being displaced from the NE, due to the downregulation of their anchoring partners (e.g., Lamins and SUNs). The observed inhibitory effects on 2D migration and cell polarity upon PDI inhibition further support the hypothesis of a disruption in the LINC complex. This disruption is corroborated by phenocopying these cellular effects when specific LINC complex constituents are depleted [70,71]. Furthermore, the cellular phenotypes described herein agree with previous PDI inhibition studies. Anti-proliferative effects, cell-substratum deficits, and impairment of wound closure, using a variety of PDI inhibitors, have been described in breast cancer cells [72]. Previous research also finds the transwell invasion and transendothelial migration properties of MDA-MB-231 cells to be compromised upon PDI inhibition [72]. However, our results diverge from this pattern, which we attribute to the utilisation of inserts featuring smaller pores (i.e., diameters of 5 μm compared to 8 μm). 

Our prevailing conceptual model is that the downregulation of IF proteins (e.g., nuclear lamins and cytoplasmic IFs) and LINC complex disruption increases the pliability of PDI-inhibited MDA-MB-231 cells/nuclei, enabling the cells to transverse through the smaller 5 μm pores (Figure 7). Several lines of evidence support this model. In melanoma cells, downregulation of Lamin A/C has been shown to increase nuclear deformability, facilitating migration through rigid pores [73]. Similarly, in breast cancer, reduced Lamin A levels have been associated with enhanced metastatic capacity, due to facilitated confined cell movement [74]. Additionally, LINC complex disruption has been found to decrease the mechanical stiffness of Swiss3T3 cells [75]. Studies on keratin-free murine epithelia have demonstrated increased cellular invasiveness and deformability [76]. Loss of K8/18 has been linked to collective cell migration of epithelia [77] and increased invasiveness of breast cancer cells [78]. Furthermore, in fibroblasts, the presence of Vimentin has been observed to maintain nuclear integrity by accommodating cellular stiffening under compression [79]. 

Disulphide bonds play fundamental roles in cytoplasmic IF structure, aiding network formation and influencing localisation [80]. In Vimentin, Cys328 is involved in intermolecular associations [81], playing profound roles in filament formation, network organisation, and dynamics [82,83]. The silencing of PDIA3 in BO2 cells (an MDA-MB-231 subclone) reorganises Vimentin around the nucleus and impairs bone metastasis in mice [84]. Thus, it is intriguing that, considering 16F16’s preference for inhibiting PDIA3, we observed a reduction in perinuclear Vimentin aggregation in 16F16-treated cells, but not in PACMA31-treated cells. This evidence also suggests that the underlying molecular mechanisms leading to increased cell pliability and restrictive migration may differ between 16F16 and PACMA31 treatments, reflecting their different off-target effects. In agreement, PDIA1 is known to directly associate with β-actin Cys374 and regulate cell adhesion [85]. The oxidation of β-actin-Cys374 reduces both F-actin polymerisation and stability [86]. It is notable that PACMA31, a more potent inhibitor of PDIA1, exhibited stronger effects on F-actin reorganisation to the NE compared to 16F16. However, it is also established that 16F16 treatment diminishes stress-fiber formation, altering the attachment and cell morphology of MDA-MB-231 cells [39]. It is plausible that the increase in perinuclear F-actin structures in PDI-inhibited MDA-MB-231 cells is potentially a cellular stress response, aimed at compensating for heightened nuclear pliability and the reduction of Nesprin-2 at the NE. Further investigation is needed to reveal the molecular mechanisms underlying F-actin reshuffling, IF restructuring, and their contribution to cell/nuclear biomechanics upon PDI inhibition. 

## 5. Conclusions

The data of the current study reveal additional complexity in the spatial arrangement and molecular composition of SUN-domain proteins across the nucleus of non-invasive MCF10A breast epithelia and MDA-MB-231 breast cancer cells. The hypothesised enhanced SUN2 network connectivity at the nuclear periphery of MCF10A cells suggests potential adherence to the “form follows function” principle by D’Arcy Wentworth Thompson, reinforcing the structural integrity of the nuclear region proximal to the cytoskeleton-rich cytoplasm and cell-substratum adhesion sites. Similarly, the absence of disulphide bridges in SUN2 and Lamin B1 within MDA-MB-231 cells may weaken their LINC complex networks, potentially contributing to their invasive phenotype. In agreement, pharmacological inhibition of PDIs results in the downregulation of SUN-domain and Lamin proteins in MDA-MB-231 cells, thereby enhancing cell invasiveness in confined spaces. This underscores the necessity for meticulously designed therapeutic strategies targeting members of the PDI family, as their inhibition may bolster, rather than inhibit as intended, the metastatic potential of tumour cells in vivo. 

## Data Availability

The raw data supporting the conclusions of this article will be made available by the authors on request.

## Supplementary Materials

The following supporting information can be downloaded at: https://www.mdpi.com/article/10.3390/cells13110906/s1, Figure S1: The staining pattern of SUN2 exhibits an asymmetric distribution between the central and peripheral nuclear regions; Figure S2: The expression and subcellular localisation of PDIA1 is similar in MCF10A and MDA-MB-231 cells; Figure S3: MTT assay of MDA-MB-231 cells exposed to various concentrations of PDI inhibitors in the presence (complete media) and absence of serum proteins (serum-free media); Figure S4: InstantBlue Coomassie staining of polyacrylamide gel validates the presence of equal amounts of MDA-MB-231 cellular proteins; Figure S5: PACMA31 treatment, but not 16F16 treatment, reduces the expression levels of Lamin B1 in MDA-MB-231 cells; Figure S6: PDI inhibition results in the formation of perinuclear F-actin structures in MDA-MB-231 cells; Figure S7: The expression levels of keratin, β-tubulin, and β-actin are reduced in PACMA31-treated MDA-MB-231 cells.; Figure S8: PDI inhibition reduces MDA-MB-231 cell migration; Table S1: Primary antibodies for immunofluorescence staining; Table S2: Secondary antibodies/fluorescent stains for immunofluorescence staining; Table S3: Primary antibodies for Western blotting; Table S4: Secondary antibodies for Western blotting.
