# Supplementary material for: Inhibition of PDIs Downregulates Core LINC Complex Proteins, Promoting the Invasiveness of MDA-MB-231 Breast Cancer Cells in Confined Spaces In Vitro
Source: Cells. 2024 May 24;13(11):906. doi: 10.3390/cells13110906 (PMC11172124; doi:10.3390/cells13110906)
Supplement: Supplementary file 1 [file cells-13-00906-s001.zip › Antibody tables PDI Final.pdf]

Table S1. Primary antibodies for immunofluorescence staining

Antibody	Supplier	Product Code	Species	Dilution
SUN1	Abcam	Ab124770	Rabbit	1:200
SUN2	Abcam	Ab124916	Rabbit	1:200
HO-1	Proteintech	10701-1-AP	Rabbit	1:200
PDIA1	Abcam	Ab2792	Mouse	1:100
Nesprin-2 giant	Abcam	Ab204308	Rabbit	1:40
pAbK1	In-house	-	Rabbit	1:100
Vimentin	In-house	-	Rabbit	1:400
Keratin 8/18	Abcam	Ab17139	Mouse	1:500
GM-130	BD Biosciences	610823	Mouse	1:200

Table S2. Secondary antibodies/ fluorescent stains for immunofluorescence staining

Antibody	Supplier	Product Code	Species	Dilution
Anti-Rabbit (Alexa Fluor™ 568)	Invitrogen	A11036	Goat	1:1,000
Anti-Mouse (Alexa Fluor™ 568)	Invitrogen	A11031	Goat	1:1,000
Anti-Mouse (Alexa Fluor™ 488)	Invitrogen	A21202	Donkey	1:1,000
Fluorescent stains				Concentration
TRITC-Phalloidin	Tocris	5783	-	20 ng/mL
DAPI	Sigma-Aldrich	62247	-	2 µg/mL

Table S3. Primary antibodies for Western blotting

Antibody	Supplier	Product Code	Species	Dilution
SUN1	Abcam	Ab124770	Rabbit	1:1,000
SUN2	Abcam	Ab124916	Rabbit	1:1,000
Beta-Tubulin	Proteintech	66240-1-Ig	Mouse	1:5,000
Beta-Actin	Sigma-Aldrich	A5316	Mouse	1:5,000
HO-1	Proteintech	10701-1-AP	Rabbit	1:1,000
PDIA1	Abcam	Ab2792	Mouse	1:1,000
Lamin A/C (Jol2)	In-house	-	Mouse	1:40
Lamin B1	Abcam	Ab16048	Rabbit	1:3,000
GAPDH	Proteintech	60004-1-Ig	Mouse	1:10,000
Vimentin	In-house	-	Rabbit	1:400
Keratin 8/18	Abcam	Ab17139	Mouse	1:1,000
Pan-keratin	Sigma-Aldrich	MAB3412	Mouse	1:1,000

Table S4. Secondary antibodies for Western blotting

Antibody	Supplier	Product Code	Species	Dilution
Anti-Rabbit POD	Invitrogen	31460	Goat	1:5,000
Anti-Mouse POD	Invitrogen	31430	Goat	1:10,000
