# Supplementary material for: Inhibition of PDIs Downregulates Core LINC Complex Proteins, Promoting the Invasiveness of MDA-MB-231 Breast Cancer Cells in Confined Spaces In Vitro
Source: Cells. 2024 May 24;13(11):906. doi: 10.3390/cells13110906 (PMC11172124; doi:10.3390/cells13110906)
Supplement: Supplementary file 1 [file cells-13-00906-s001.zip › Young et al 2024 05 07 Revised supplement.pdf]

Supplemental Material:

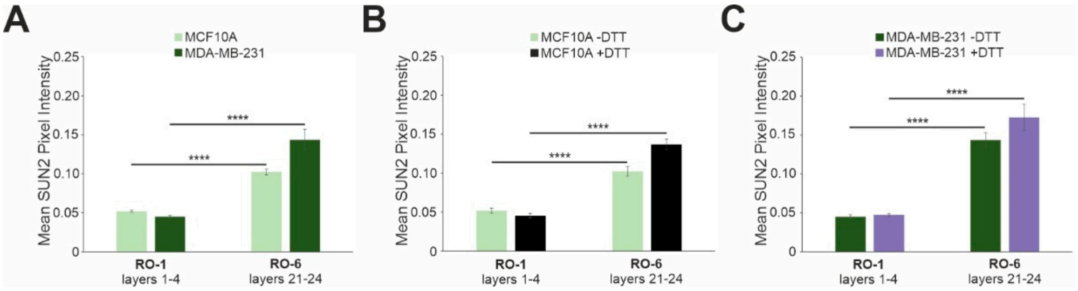

Supplement Figure S1. The staining pattern of SUN2 exhibits an asymmetric distribution between the central and peripheral nuclear regions. (A) The average SUN2 pixel intensity in the central nuclear region (Region of Interest [RO-1]) and peripheral nuclear region (RO-6) of MCF10A and MDA-MB-231 cells is shown. The error bars depict the Standard Error of the Mean (SEM), $n \geq 5$. Statistical significance was assessed using Student's unpaired t-test; the asterisks (****) denotes significance $p \leq 0.0001$. **(B)** The average SUN2 pixel intensity in the presence (+) and absence (-) of 1 µM DTT in the central (RO-1) and peripheral (RO-6) regions of MCF10A nuclei is shown. The error bars represent the Standard Error of the Mean (SEM), $n \geq 5$. Statistical significance (****, $p \leq 0.0001$) was assessed using a Student's unpaired t-test. **(C)** The average SUN2 pixel intensity in the presence (+) and absence (-) of 1 µM DTT in the central (RO-1) and peripheral (RO-6) regions of MDA-MB-231 nuclei is depicted. The error bars represent the Standard Error of the Mean (SEM), $n \geq 5$. Statistical significance (****, $p \leq 0.0001$) was assessed using Student's unpaired t-test.

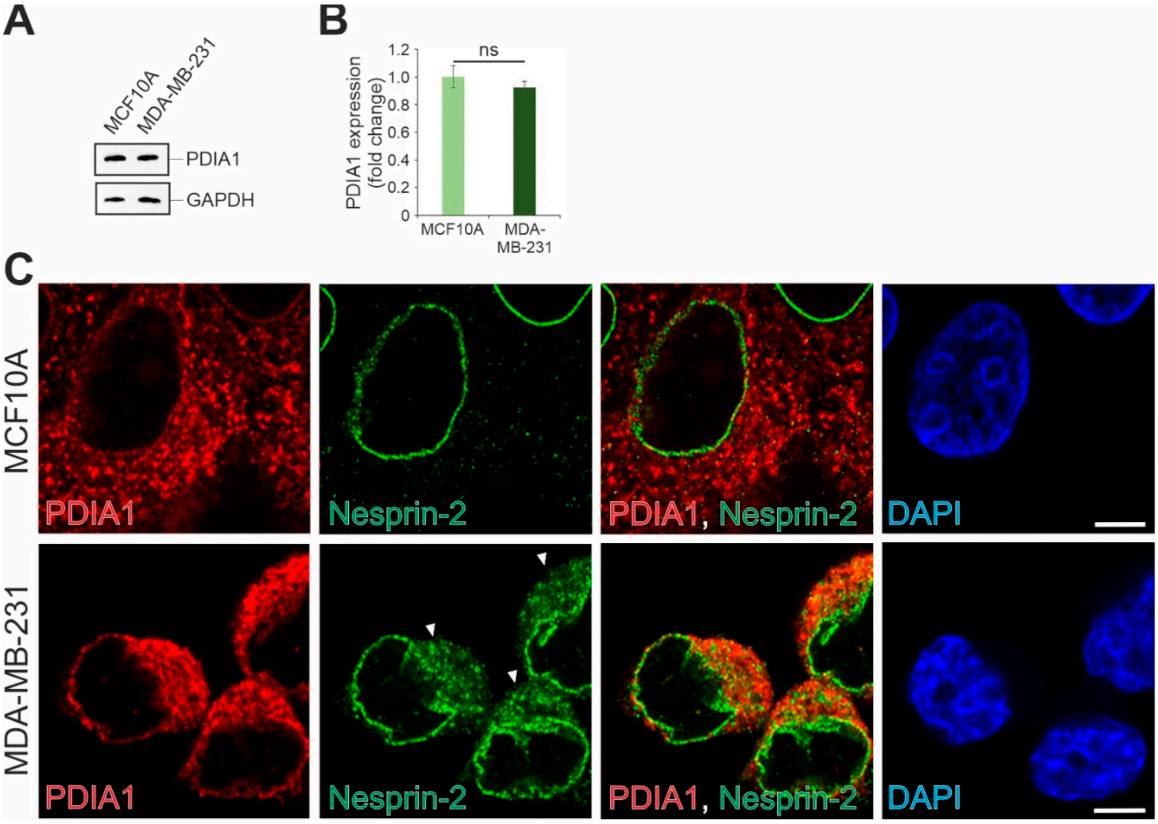

Supplement Figure S2. The expression and subcellular localisation of PDIA1 is similar in MCF10A and MDA-MB-231 cells. (**A**) Western blot analysis of PDIA1 expression in MCF10A and MDA-MB-231 cells. GAPDH expression acts as a protein loading control. (**B**) Quantitative analysis of PDIA1 protein expression in MCF10A and MDA-MB-231 cells. Data are represented as the mean ±SEM, $n = 3$. Statistical significance was assessed using a

Student's unpaired t-test; "ns", non significant. (**C**) Confocal microscopy imaging of PDIA1 and Nesprin-2 (pAbK1) immunofluorescence staining. DAPI is utilised for nuclear staining. Scale bar: 5 µm.

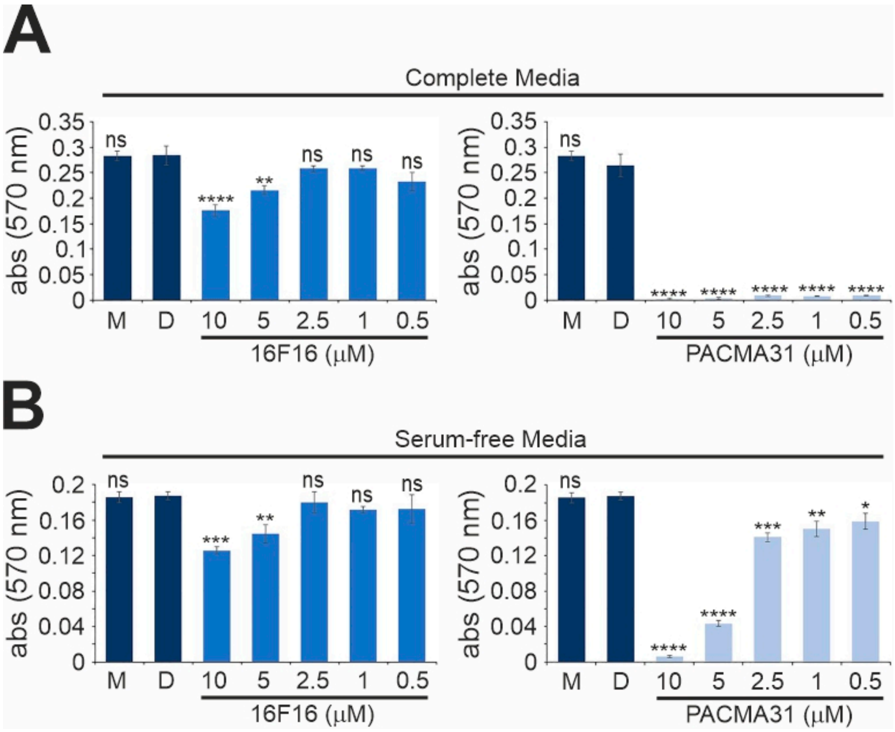

Supplement Figure S3. MTT assay of MDA-MB-231 cells exposed to various concentrations of PDI inhibitors in the presence (complete media) and absence of serum proteins (serum-free media). (A) MTT assay results of PDI inhibited MDA-MB-231 cells using complete media. The data represent the mean ±SEM, n = 3. Statistical significance (** $p \leq 0.01$, **** $p \leq 0.0001$) was assessed using a one-way ANOVA with a Dunnett's post-hoc test, in comparison to the vehicle treatment (DMSO; [D]). Non-significant differences are denoted as "ns". **(B)** MTT assay results of PDI inhibited MDA-MB-231 cells using serum-free media. The data represent the mean ±SEM, n = 3. Statistical significance (indicated by *) was assessed using one-way ANOVA with a Dunnett's post-hoc test, in comparison to the vehicle treatment (DMSO; [D]). Non-significant differences are denoted as "ns", * $p \leq 0.05$, ** $p \leq 0.01$, *** $p \leq 0.001$, **** $p \leq 0.0001$.

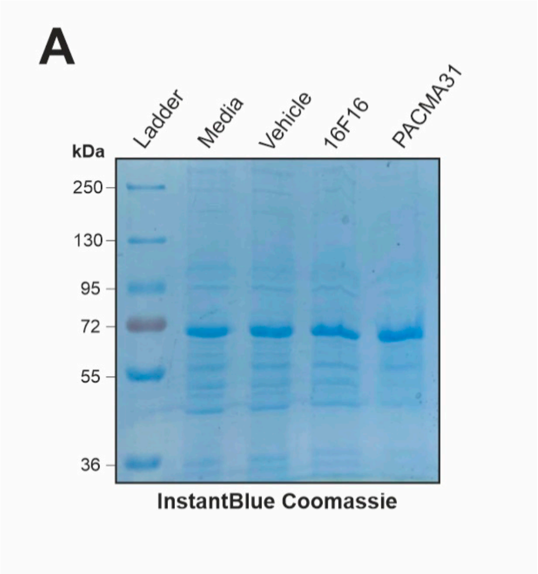

Supplement Figure S4. InstantBlue Coomassie staining of polyacrylamide gel validates the presence of equal amounts of MDA-MB-231 cellular proteins. Cell lysates were obtained from MDA-MB-231 cells exposed to media alone, DMSO (vehicle), 16F16, or PACMA31 treatment. The samples were analysed using SDS-PAGE and then stained with InstantBlue Coomassie.

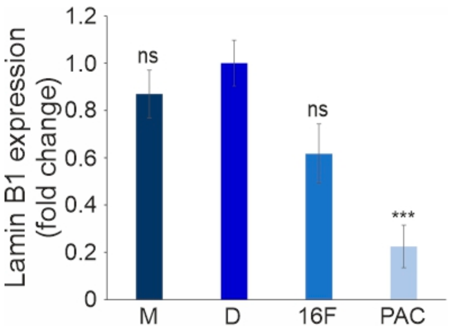

Supplement Figure S5. PACMA31 treatment but not 16F16 treatment, reduces the expression levels of Lamin B1 in MDA-MB-231 cells. Western blot analysis of Lamin B1 protein levels in MDA-MB-231 cells treated with media (M), DMSO (D), 16F16 (16F), and PACMA31 (PAC). The data represent the mean ±SEM, $n = 3$. The statistical significance (indicated by *) was assessed using a one-way ANOVA with a Dunnett's post-hoc test, in comparison to the vehicle treatment (DMSO; [D]). Non-significant differences are denoted as "ns", *** $p \leq 0.001$.

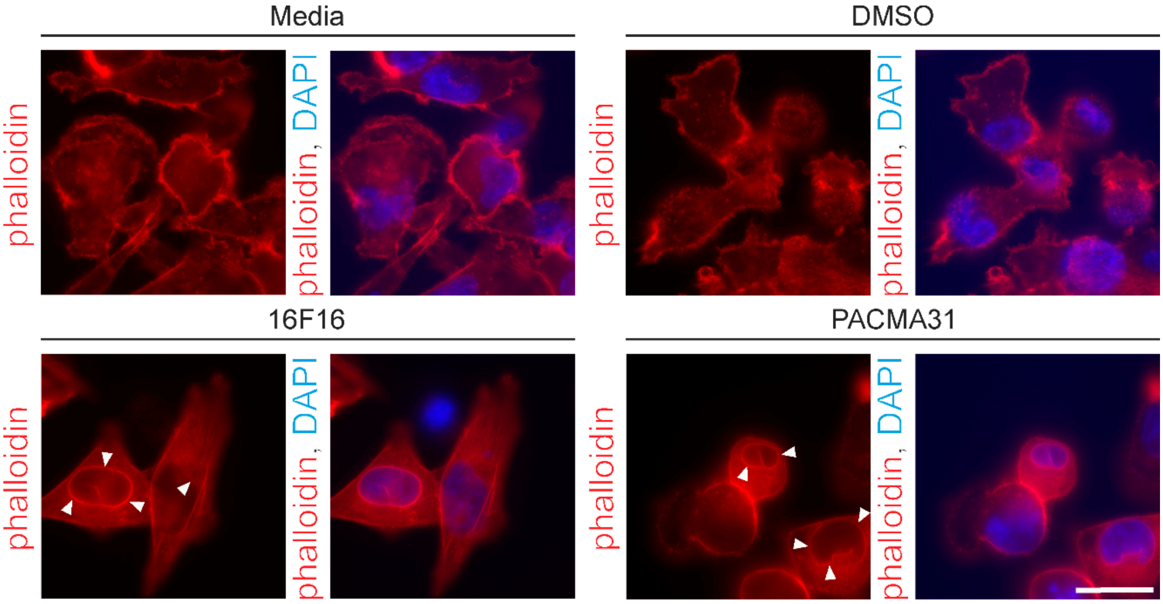

Supplement Figure S6. PDI inhibition results in the formation of perinuclear F-actin structures in MDA-MB-231 cells. Epifluorescence microscopy examination of TRITC-phalloidin (labels F-actin) and DAPI (labels the nucleus) stained MDA-MB-231 control (Media and DMSO) cells relative to PDI inhibited cells. The white arrowheads denote perinuclear F-actin structures. Scale bar: 20 µm.

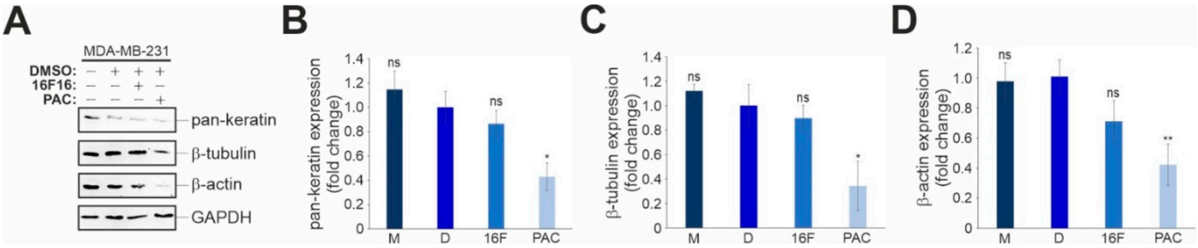

Supplement Figure S7. The expression levels of keratin, β-tubulin and β-actin are reduced in PACMA31 treated MDA-MB-231 cells. (**A**) Western blot analysis of media (M), DMSO (D), 16F16, and PACMA31 (PAC) treated cells using the indicated antibodies. GAPDH highlights the equal loading of protein lysates. **(B-D)** Quantitative examination of pan-keratin (**B**), β-tubulin (**C**) and β-actin (**D**) protein levels. The error bars represent the mean ±SEM, n = 3. The statistical significance (indicated by *) was assessed using a one-way ANOVA with a Dunnett's post-hoc test, with comparisons made to the vehicle treatment (DMSO; [D]). Non-significant differences are denoted as "ns", * $p \leq 0.05$, ** $p \leq 0.01$.

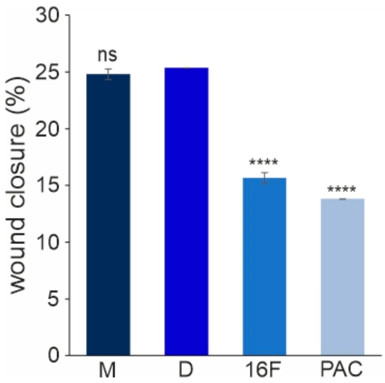

Supplement Figure S8. PDI inhibition reduces MDA-MB-231 cell migration. The graph indicates the wound closing capacity of MDA-MB-231 cells treated with media (M), DMSO (D), 16F16, and PACMA31 (PAC), 6 hours post-scratch wound induction. The error bars represent the mean ±SEM, n = 3. The statistical significance (**** $p \leq 0.0001$) was assessed using a one-way ANOVA with a Dunnett's post-hoc test, with comparisons made to the vehicle treatment (DMSO; [D]). Non-significant differences are denoted as "ns".
